# Identifying Bioactive Ingredients and Antioxidant Activities of Wild *Sanghuangporus* Species of Medicinal Fungi

**DOI:** 10.3390/jof9020242

**Published:** 2023-02-11

**Authors:** Hao Wang, Jin-Xin Ma, Dong-Mei Wu, Neng Gao, Jing Si, Bao-Kai Cui

**Affiliations:** 1 Institute of Microbiology, School of Ecology and Nature Conservation, Beijing Forestry University, Beijing 100083, China; 2 Xinjiang Academy of Agricultural and Reclamation Sciences/Xinjiang Production and Construction Group Key Laboratory of Crop Germplasm Enhancement and Gene Resources Utilization, Biotechnology Research Institute, Shihezi 832000, China

**Keywords:** medicinal fungi, fungal resources, *Sanghuangporus*, bioactive ingredient, antioxidant activity

## Abstract

*Sanghuangporus* refers to a group of rare medicinal fungi with remarkable therapeutic properties. However, current knowledge on the bioactive ingredients and antioxidant activities of different species of this genus is limited. In this study, a total of 15 wild strains from 8 species of *Sanghuangporus* were selected as the experimental materials for identification of the bioactive components (polysaccharide, polyphenol, flavonoid, triterpenoid, and ascorbic acid) and antioxidant activities (scavenging activities against hydroxyl, superoxide, DPPH, and ABTS radicals; superoxide dismutase activity; and ferric reducing ability of plasma). Notably, individual strains contained different levels of various indicators, among which *Sanghuangporus baumii* Cui 3573, *S. sanghuang* Cui 14419 and Cui 14441, *S. vaninii* Dai 9061, and *S. zonatus* Dai 10841 displayed the strongest activities. The correlation analysis of bioactive ingredients and antioxidant activities revealed that the antioxidant capacity of *Sanghuangporus* is mainly associated with the contents of flavonoid and ascorbic acid, followed by polyphenol and triterpenoid, and finally, polysaccharide. Together, the results obtained from the comprehensive and systematic comparative analyses contribute further potential resources and critical guidance for the separation, purification, and further development and utilization of bioactive agents from wild *Sanghuangporus* species, as well as the optimization of their artificial cultivation conditions.

## 1. Introduction

Sanghuang was first recorded more than two thousand years ago in *Shen Nong Materia Medica*, the earliest monograph on traditional Chinese medicine [1,2]. As a group of rare medicinal fungi, this species was widely used for treating gynecological diseases, protecting liver and stomach, expelling toxins, and achieving other therapeutic purposes [3,4]. The earliest modern research on the beneficial properties of sanghuang was conducted by the group working with the Japanese scholar Tetsuro Ikekawa in 1968 [5], which documented an inhibitory rate of sanghuang on the mouse sarcoma cell line, S-180, of up to 96.7%. Since then, sanghuang has attracted extensive attention due to its potential utility in functional foods, biomedical products, and the drug industry. In recent years, multifarious bioactive compounds have been identified in sanghuang, including polysaccharides, polyphenols, flavonoids, triterpenoids, and ascorbic acids (AAs) [6,7,8,9]. In addition to anti-tumor activity, numerous studies have confirmed potent antioxidant, antimicrobial, anti-inflammatory, hepatoprotective, anti-diabetic, and immunological properties of sanghuang [10,11,12,13,14]. For instance, Liu et al. [15] reported that the ethyl acetate extract of *Inonotus sanghuang* exhibits strong antioxidant capacity, antiproliferative effects against prostate cancer cells, and antimicrobial activities against three Gram-positive bacterial species. Yoo et al. [16] demonstrated an immunomodulatory effect of extracts of *Phellinus baumii* in preventing an immunosuppressive state following oral administration. An exopolysaccharide purified from *S. sanghuang* was shown to possess antitumor activities against human hepatoma and breast cancer cells [17]. Four polyphenols isolated by Zhang et al. [18] from the fermentation products of *S. sanghuang* exhibited significant antioxidant activity, ranging from 5.310 to 23.110 μmol/L, in human hepatoma cells. A polysaccharopeptide separated from *S. lonicericola* harbors strong concentration-dependent antioxidant ability against hydroxyl, superoxide, and DPPH radicals, as well as total reducing power activity in vitro [19].

For a long time, sanghuang is generally thought to be comprised of multiple species in the genera of *Phellinus* and *Inonotus* (family Hymenochaetaceae), which are distinctive by their yellow-brown and hard fruiting bodies [20,21,22,23]. In 2016, a new genus, *Sanghuangporus*, branching from the family Hymenochaetaceae, was established by Zhou et al. [24]. Subsequently, a new species from tropical Taiwan, China, *S. vitexicola*, was reported [25]. To date, a total of 18 species have been described in the genus *Sanghuangporus*, 11 of which are distributed in China, specifically, *S. alpinus*, *S. baumii*, *S. lonicericola*, *S. quercicola*, *S. sanghuang*, *S. subbaumii*, *S. toxicodendri*, *S. vaninii*, *S. vitexicola*, *S. weigelae*, and *S. zonatus* [23,25,26]. Notably, some species are endemic to specific areas and form strict parasitic relationships with their host plants [27,28]. Earlier studies on the metabolites and bioactivities of a number of *Sanghuangporus* species, such as *S. baumii*, *S. lonicericola*, *S. sanghuang*, and *S. vaninii*, have been conducted [16,17,29,30]. Nevertheless, a comparison of the discrepancies in activity among different strains is a challenge due to variable testing methods and experimental operations. At present, the absence of research on a number of *Sanghuangporus* species and the lack of systematic comparisons limit the screening and utilization of strains with potent therapeutic activity.

Since oxidative damage is associated with the occurrence of multiple diseases, such as cancer, aging, and inflammation [2,31], an investigation of the antioxidant activities of *Sanghuangporus* species could provide significant therapeutic benefits. Due to their unique growth mechanisms, stringent relationships with host plants, and specific locations of occurrence, wild *Sanghuangporus* species are relatively rare [23,27,28]. For example, *S. sanghuang* only grows on *Morus*, and as a result, most species have not been successfully cultivated. Submerged fermentation is commonly used to produce bioactive ingredients, owing to the considerable advantages of shorter incubation time and reduced risk of contamination [32]. The main objective of the present study was to conduct a comprehensive and systematic comparison of the bioactivities of multiple species and strains of wild *Sanghuangporus* through an analysis of their active constituents and antioxidant indexes in fermentation broth. The results should bridge the gap in knowledge regarding the bioactivities of *Sanghuangporus* species, provide critical guidance for screening strains with improved activities, and further aid in optimizing the separation, purification, development, and utilization of bioactive derivatives as well as their artificial cultivation conditions.

## 2. Materials and Methods

### 2.1. Materials

The 15 wild strains from 8 species of *Sanghuangporus* examined in this study and their properties are summarized in detail in Table 1. All strains were isolated from wild fruiting bodies and cultured on potato dextrose agar (PDA) slants for 10 days at 28 °C. The strains were sub-cultured once every two months, maintained at 4 °C, and subsequently deposited at the Beijing Forestry University.

### 2.2. Chemicals and Reagents

Glucose, phenol, sulfuric acid, ethanol, pyrogallol, Folin–Ciocalteu reagent, Na_2_CO_3_, ethyl acetate, rutin, NaNO_2_, Al(NO_3_)_3_, NaOH, oleanolic acid, acetic acid, vanillin, perchloric acid, trichloromethane, 2,2′-azino-bis(3-ethylbenzthiazoline-6-sulfonic acid) (ABTS), potassium persulfate, sodium acetate, 2,4,6-tris(2-pyridyl)-s-triazine (TPTZ), hydrochloric acid, FeCl_3_, FeSO_4_, and ferrozine were of analytical grade. The assay kits for AA content; scavenging activities against hydroxyl, superoxide, and 1,1-diphenyl-2-picrylhydrazyl (DPPH) radicals; and superoxide dismutase (SOD) activity were purchased from the Nanjing Bioengineering Institute (Nanjing, China).

### 2.3. Molecular Identification

All the strains used were identified based on molecular analysis of internal transcribed spacer (ITS) rDNA. Total genomic DNA was extracted using an improved CTAB protocol [33], and ITS regions were amplified via PCR using the primers ITS5 (5′-GGAAGTAAAAGTCGTAACAAGG-3′) and ITS4 (5′-TCCTCCGCTTATTGATATGC-3′). Thereafter, the PCR products were sent to the Beijing Genomics Institute (Beijing, China) for sequencing and aligned in the National Center for Biotechnology Information (NCBI) database (https://www.ncbi.nlm.nih.gov/).

### 2.4. Submerged Fermentation and Sample Preparation

For submerged fermentation, the isolates were transferred from the agar slants to yeast dextrose agar (YDA) Petri dishes (g/L distilled water: glucose 20, yeast extract 5, KH_2_PO_4_ 1.0, MgSO_4_∙7H_2_O 0.5, agar 35, and vitamin B1 0.01). After activation at 28 °C for 10 days, five 10 mm^2^ mycelial discs of each agar culture were cut with a sterilized knife and immediately inoculated into a 250 mL flask with 100 mL of yeast dextrose medium (YD, identical to YDA without agar). The seed culture was grown in a rotary shaker at 150 rpm and 28 °C for 10 days and homogenized using a modular homogenizer at 5000 rpm for 30 s. A 10 mL aliquot of the prepared seed cultures was inoculated into a 250 mL flask containing 100 mL of YD medium and incubated at 28 °C on a rotary shaker at 150 rpm for 14 days. During the 14-day submerged fermentation period, the samples were harvested every 2 days via centrifugation at 4000 rpm for 20 min and subjected to filtration. The resulting mycelia were washed with distilled water four times, dried to a constant weight at 65 °C, and weighed for biomass yield. The obtained cell-free fermentation broth was used to determine the following activity indicators: contents of polysaccharide, polyphenol, flavonoid, triterpenoid, and AA; scavenging activities against hydroxyl, superoxide, DPPH, and ABTS radicals; SOD activity; and ferric reducing ability of plasma (FRAP).

### 2.5. Measurement of Bioactive Ingredients

The phenol-sulfuric acid method was used to measure the polysaccharide contents of the 15 wild strains from 8 species of *Sanghuangporus* at a wavelength of 595 nm with _D_-glucose as a standard [34,35]. The regression equation of the standard curve is *y* = 23.7500*x* + 1.2352, with a correlation coefficient *R*^2^ of 0.9969. Deionized water, instead of the sample, and phenol were set as the blank and control groups, respectively. The polysaccharide content of each sample was calculated as the equivalent of glucose according to the standard curve.

The polyphenol contents were determined using the Folin–Ciocalteu method at 750 nm with pyrogallol as a standard [36,37]. The regression equation of the standard curve is *y* = 2.7622*x* + 0.0123, with a correlation coefficient *R*^2^ of 0.9971. Deionized water, instead of the sample, and the Folin–Ciocalteu reagent were set as the blank and control groups, respectively. The polyphenol content of each sample was calculated as the equivalent of pyrogallol according to the standard curve.

An assay of the flavonoid content was conducted using the method of Zhu et al. [38,39] based on a colorimetric measurement at 510 nm. The regression equation of the standard curve is *y* = 0.0166*x* − 0.0360, with a correlation coefficient *R*² of 0.9910. Ethanol at a 70% (*v*/*v*) concentration, instead of the sample, and a rutin aqueous solution were set as the blank and control groups, respectively. The flavonoid content of each sample was calculated as the equivalent of rutin according to the standard curve.

The triterpenoid contents of the 15 wild *Sanghuangporus* strains were estimated according to the method of Baby et al. [40]. Increased absorbance was monitored at 550 nm based on the oxidization of the oleanolic acid substrate. The regression equation of the standard curve is *y* = 0.0336*x* + 0.0499, with a correlation coefficient *R²* of 0.9978. Deionized water, instead of the sample, and oleanolic acid were set as the blank and control groups, respectively. The triterpenoid content of each sample was calculated as the equivalent of oleanolic acid based on the standard curve.

The AA contents were assessed based on a colorimetric method in accordance with the manufacturer’s instructions (Nanjing, China).

### 2.6. Determination of Antioxidant Activities

The protocol for determining the hydroxyl radical scavenging activities of the *Sanghuangporus* species was based on a colorimetric method and implemented following the manufacturer’s instructions (Nanjing, China). One unit (U/mL) was defined as the amount of the sample required to reduce the concentration of H_2_O_2_ in the reaction system by 1 mM per minute per milliliter at 37 °C.

Scavenging activities against superoxide radicals were quantified in accordance with the manufacturer’s instructions (Nanjing, China). One unit (U/L) was defined as the amount of the sample required to suppress superoxide radicals in the reaction system per liter at 37 °C in 40 min.

The scavenging activities of the 15 wild *Sanghuangporus* strains against DPPH radicals were examined using a colorimetric method following the manufacturer’s instructions (Nanjing, China).

ABTS radical scavenging activities were determined by measuring absorbance changes at 734 nm according to the method of Miller et al. [41]. The following formula was used: ABTS radical scavenging activity (%) = [1 − (*A_sample_* − *A_control_*)/*A_blank_*] × 100, whereby *A_sample_* and *A_blank_* represent the absorbance values of the ABTS^+^ solution with or without the sample, and *A_control_* is the absorbance value of deionized water instead of the ABTS solution. Rutin was selected as the positive reference.

The hydroxylamine method was used as the experimental strategy for estimating SOD activity as recommended by the manufacturer (Nanjing, China). One unit (U/mL) was defined as the amount of the sample required to inhibit the rate of superoxide radicals to 50% in the reaction system per milliliter at 37 °C in 40 min.

FRAP is the capacity to reduce ferric tripyridyl triazine to ferrous triazine. It was determined using the method of Benzie and Strain [42] based on the measurement of absorbance changes at 593 nm. A FRAP working solution was freshly prepared by mixing 25.0 mL of 0.3 M acetic acid-sodium acetate buffer (pH 3.6), 2.5 mL of 0.01 M TPTZ solution, and 2.5 mL of 0.02 M FeCl_3_ solution. The regression equation of the standard curve is *y* = 0.0047*x* + 0.1363, with a correlation coefficient *R^*2*^* of 0.9978. The absorbance of the mixture at 593 nm was measured as *A_sample_*. Deionized water, instead of the sample, and the FRAP working solution were set as *A_blank_* and *A_control_*, respectively. The FRAP of each sample was calculated based on the concentration of FeSO_4_ equivalent corresponding to the value of *A_sample_* − *A_blank_* − *A_control_* according to the standard curve.

### 2.7. Data Analysis

All results are presented as means ± standard deviations of three independent experiments. Comparison of any two groups was conducted with one-way analysis of variance (ANOVA) followed by the Waller–Duncan test using the SPSS 20.0 software. Correlations between groups were analyzed using the unary linear regression model in the R 4.0.3 software and mapped through the “BasicTrendline”, “ggplot2”, and “patchwork” packages in R studio. Differences were considered significant and highly significant at *p* < 0.05 and *p* < 0.01, respectively.

## 3. Results and Discussion

### 3.1. Identification of Wild Sanghuangporus Strains

All isolates were identified based on a molecular analysis of the ITS regions via genomic DNA extraction and PCR amplification. Based on a comparison with the ITS-rDNA sequences in the GenBank database, the strains were identified as follows: *S. alpinus* (strain numbers Cui 12444, Cui 12456, and Cui 17052), *S. baumii* (Cui 3573 and Dai 13331), *S. lonicericola* (Dai 8375 and Dai 17304), *S. quercicola* (Wei 7575), *S. sanghuang* (Cui 14419 and Cui 14441), *S. vaninii* (Dai 8236, Dai 8245, and Dai 9061), *S. weigelae* (Dai 15768), and *S. zonatus* (Dai 10841). All sequences were deposited in GenBank (Accession numbers: OP962409–OP962417).

### 3.2. Mycelial Biomass

In the early stages of submerged fermentation, mycelial pellets derived from the wild strains were small, and the fermentation broths were clear and transparent. During the course of adaptation of these strains to the environment, the mycelial pellets became dense and full and began to secrete polysaccharides, polyphenols, and other molecules, resulting in the yellowish-brown color of the fermentation broths. At the late stages, the mycelial pellets were crowded and occasionally underwent autolysis. Moreover, the fermentation broths displayed a muddy color, presumably due to pigment accumulation.

**Figure 1:**
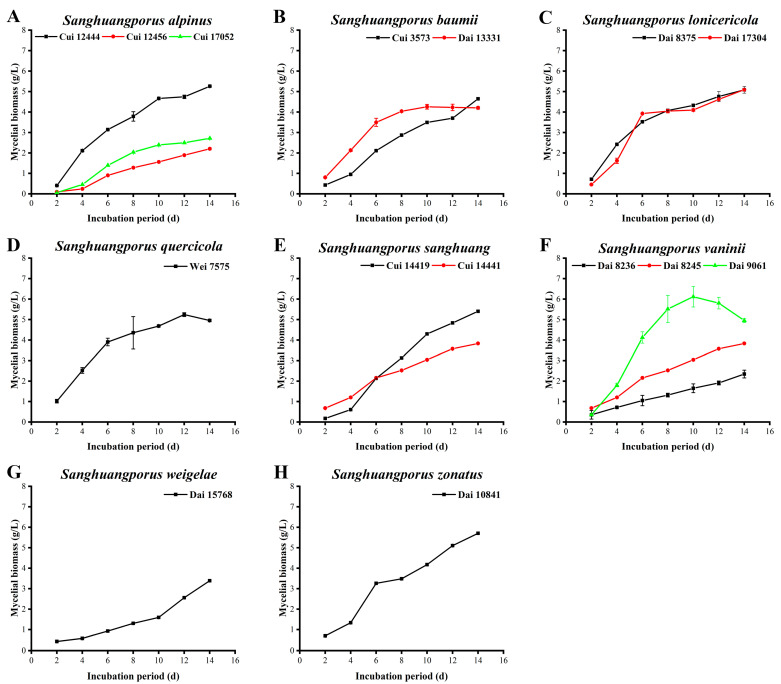
Variations in mycelial biomass during submerged fermentation of 15 wild strains from 8 species of *Sanghuangporus*. (**A**) *Sanghuangporus alpinus* Cui 12444, Cui 12456, and Cui 17052. (**B**) *Sanghuangporus baumii* Cui 3573 and Dai 13331. (**C**) *Sanghuangporus lonicericola* Dai 8375 and Dai 17304. (**D**) *Sanghuangporus quercicola* Wei 7575. (**E**) *Sanghuangporus sanghuang* Cui 14419 and Cui 14441. (**F**) *Sanghuangporus vaninii* Dai 8236, Dai 8245, and Dai 9061. (**G**) *Sanghuangporus weigelae* Dai 15768. (**H**) *Sanghuangporus zonatus* Dai 10841.

The mycelial biomass of all strains was measured every two days during the 14-day submerged fermentation period (Figure 1). Apart from *S. baumii* Dai 13331, the mycelial biomass from the majority of strains continuously increased. *Sanghuangporus vaninii* Dai 9061 and *S. quercicola* Wei 7575 achieved a maximal mycelial biomass on days 10 and 12, followed by a decrease. The highest mycelial biomass of 6.116 ± 0.499 g/L was documented for *S. vaninii* Dai 9061 on day 10. Similarly, Wang et al. [29] measured the mycelial biomass of *S. lonicericola* and *S. quercicola* under submerged fermentation, with maxima of 4.83 and 7.03 g/L on day 14, respectively.

### 3.3. Polysaccharide Content

**Figure 2:**
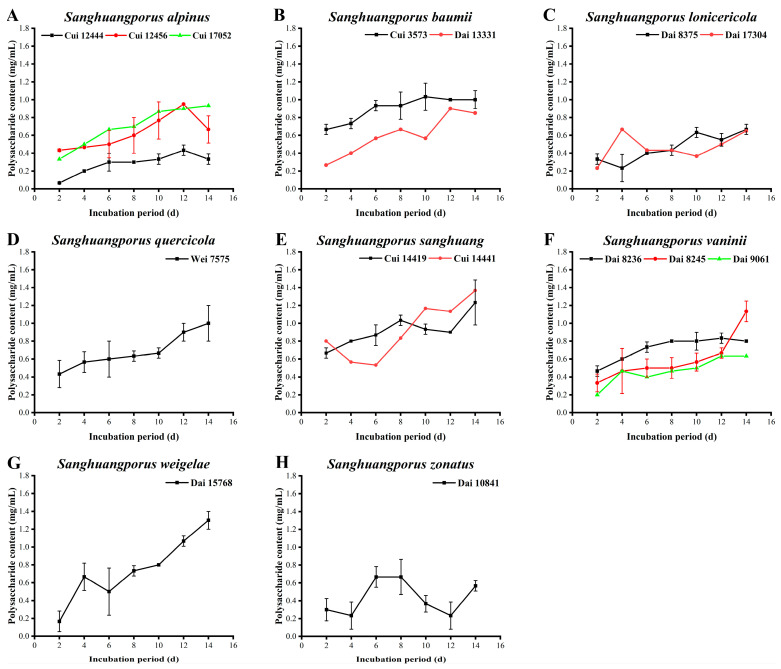
Variations in polysaccharide contents during submerged fermentation of 15 wild strains from 8 species of *Sanghuangporus*. (**A**) *Sanghuangporus alpinus* Cui 12444, Cui 12456, and Cui 17052. (**B**) *Sanghuangporus baumii* Cui 3573 and Dai 13331. (**C**) *Sanghuangporus lonicericola* Dai 8375 and Dai 17304. (**D**) *Sanghuangporus quercicola* Wei 7575. (**E**) *Sanghuangporus sanghuang* Cui 14419 and Cui 14441. (**F**) *Sanghuangporus vaninii* Dai 8236, Dai 8245, and Dai 9061. (**G**) *Sanghuangporus weigelae* Dai 15768. (**H**) *Sanghuangporus zonatus* Dai 10841.

Polysaccharides, which are macromolecular polymers composed of multiple aldoses or ketoses linked by glycosidic bonds, possess a broad spectrum of beneficial pharmacological properties, including anti-tumor, antioxidant, anti-bacterial, anti-inflammatory, and immunomodulatory activities [43,44,45,46,47,48,49]. The polysaccharide yields determined for the 15 wild strains from 8 species of *Sanghuangporus* are presented in Figure 2 and Supplementary Table S1.

The polysaccharide contents of the three wild *S. alpinus* strains are illustrated in Figure 2A. The yields of Cui 12444 and Cui 12456 display a downward trend after a continuous increase to the maximum levels of 0.433 ± 0.058 and 0.950 ± 0.231 mg/mL on day 12, respectively. Polysaccharide secretion by Cui 17052 is in an ever-growing phase during the whole cultivation period. Furthermore, the polysaccharide content of Cui 12444 is significantly lower than those of Cui 12456 and Cui 17052. As presented in Figure 2B, the polysaccharide yields of the two wild *S. baumii* strains increase with incubation time. The maximum polysaccharide levels of 1.033 ± 0.153 and 0.900 ± 0.200 mg/mL were obtained from Cui 3573 and Dai 13331 on days 10 and 12, respectively. Thereafter, a steady state or slight decrease was observed. Notably, the polysaccharide content of Cui 3573 is significantly higher than that of Dai 13331 at all stages. Song et al. [30] found that the peak level of polysaccharide in the fermentation broth of *S. baumii* was estimated to be 0.371 mg/mL on day 15, which was lower and more delayed than those of the two wild *S. baumii* strains examined in the present study. The levels of the two wild *S. lonicericola* strains are shown in Figure 2C. Except for day 2, the polysaccharide content of Dai 17304 is higher during the first eight days and lower from days 8 to 14 relative to that of Dai 8375. Both Dai 8375 and Dai 17304 display the maximum polysaccharide yields of ~0.667 ± 0.058 mg/mL, but at different time. The polysaccharide content of *S. quercicola* Wei 7575 increases continuously throughout the whole cultivation process, with a maximum value of 1.000 ± 0.200 mg/mL on day 14 (Figure 2D). The polysaccharide contents of the two wild *S. sanghuang* strains are presented in Figure 2E. Other than day 2, the content of Cui 14419 is higher than that of Cui 14441 during the first eight days but lower from days 10 to 14. The maximum yield of 1.366 ± 0.153 mg/mL was observed on day 14 for Cui 14441. As depicted in Figure 2F, the polysaccharide contents of *S. vaninii* Dai 8245 and Dai 9061 increase during the whole cultivation process, with the highest recorded values of 1.133 ± 0.115 and 0.633 ± 0.115 mg/mL, respectively, on day 14. The maximum polysaccharide yield of Dai 8236 is 0.833 ± 0.578 mg/mL on day 12, followed by a slight downward trend. Overall, the polysaccharide content of Dai 8236 is significantly higher than those of Dai 8245 and Dai 9061 in the first 12 days, but lower than that of Dai 8245 on day 14. Aside from day 4, the polysaccharide level of *S. weigelae* Dai 15768 increases continuously throughout the cultivation process, with a maximum yield (1.300 ± 0.100 mg/mL) on day 14 (Figure 2G). The polysaccharide content synthesized by *S. zonatus* Dai 10841 fluctuates from 0.233 ± 0.153 to 0.667 ± 0.196 mg/mL, with the highest value observed on day 8 (Figure 2H).

The top six strains with the highest polysaccharide yields were ranked in the following order: *S. sanghuang* Cui 14441 (1.366 ± 0.153 mg/mL) > *S. weigelae* Dai 15768 (1.300 ± 0.100 mg/mL) > *S. sanghuang* Cui 14419 (1.233 ± 0.252 mg/mL) > *S. vaninii* Dai 8245 (1.133 ± 0.115 mg/mL) > *S. baumii* Cui 3573 (1.033 ± 0.153 mg/mL) > *S. quercicola* Wei 7575 (1.000 ± 0.200 mg/mL). The majority of strains showed maximum levels on days 12 or 14. The capacity of a strain to secrete polysaccharides appears closely related to its growth status, since no remarkable differences in the appearance of peak values of mycelial biomass and polysaccharide yield were observed for most strains during the vigorous growth period. However, in recessive conditions, a strain still needs to continuously secrete secondary metabolites to promote resistance to oxidative stress at the later stages of growth, thus leading to a discrepancy in peak occurrence.

### 3.4. Polyphenol Content

**Figure 3:**
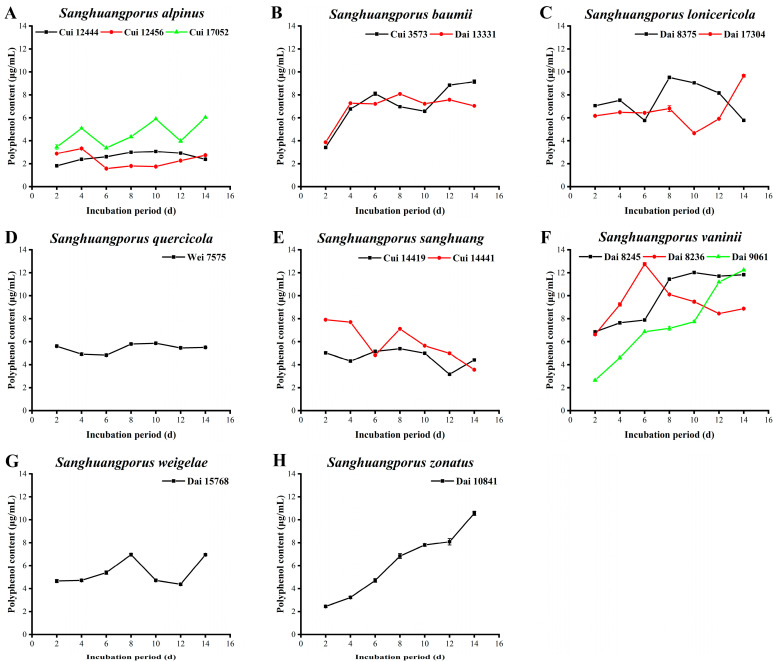
Variations in polyphenol contents during submerged fermentation of 15 wild strains from 8 species of *Sanghuangporus*. (**A**) *Sanghuangporus alpinus* Cui 12444, Cui 12456, and Cui 17052. (**B**) *Sanghuangporus baumii* Cui 3573 and Dai 13331. (**C**) *Sanghuangporus lonicericola* Dai 8375 and Dai 17304. (**D**) *Sanghuangporus quercicola* Wei 7575. (**E**) *Sanghuangporus sanghuang* Cui 14419 and Cui 14441. (**F**) *Sanghuangporus vaninii* Dai 8236, Dai 8245, and Dai 9061. (**G**) *Sanghuangporus weigelae* Dai 15768. (**H**) *Sanghuangporus zonatus* Dai 10841.

Polyphenols, which are generally composed of aromatic hydroxyl derivatives, are widely synthesized during the secondary metabolism of higher fungi. Due to the existence of hydroxyl groups on the benzene ring, which easily lose hydrogen electrons, polyphenols act as electron donors that participate in a series of reactions and thus possess multifarious biological properties, such as antioxidant and anti-tumor activities [50,51,52]. The polyphenol contents secreted by the 15 wild strains from 8 species of *Sanghuangporus* are depicted in Figure 3 and Supplementary Table S1.

The polyphenol contents of the three wild *S. alpinus* strains are presented in Figure 3A. The polyphenol yields of Cui 12444 and Cui 12456 are relatively low, with no distinct upward trend during the whole cultivation process. The polyphenol content of Cui 17052 is higher than those of Cui 12444 and Cui 12456 at all stages, with a maximum value of 6.040 ± 0.031 μg/mL on day 14. The polyphenol levels of the two wild *S. baumii* strains increase rapidly during days 2 to 4 and then fluctuate within a certain range over the last 10 days of incubation (Figure 3B). Their polyphenol contents are highest (9.154 ± 0.173 μg/mL for Cui 3573 and 8.079 ± 0.033 μg/mL for Dai 13331) on days 14 and 8, respectively. As shown in Figure 3C, polyphenol secretion by *S. lonicericola* Dai 8375 markedly decreases on day 6 and achieves a maximum of 9.523 ± 0.114 μg/mL on day 8, followed by a continuous decrease from days 8 to 14. The polyphenol derived from Dai 17304 exhibits a slight upward trend in the first eight days and decreases to a minimum on day 10. Subsequently, the level increases again, reaching a maximum of 9.663 ± 0.114 μg/mL on day 14. Interestingly, the polyphenol content of Dai 8375 is significantly higher than that of Dai 17304, except on days 6 and 14. Polyphenol secretion by *S. quercicola* Wei 7575 is relatively stable and maintains at a moderate level throughout the cultivation process (Figure 3D). The polyphenol contents of the two wild *S. sanghuang* strains roughly show a downward trend during the 14-day incubation period, with maximum values of 5.390 ± 0.023 (Cui 14419) and 7.909 ± 0.002 μg/mL (Cui 14441) on days 8 and 2, respectively (Figure 3E). Moreover, the polyphenol content of Cui 14441 is obviously higher than that of Cui 14419, except on days 6 and 14. Among the three wild *S. vaninii* strains (Figure 3F), the polyphenol yield of Dai 8236 increases to a peak level of 12.758 ± 0.133 μg/mL on day 6 and then starts to decrease. In contrast, polyphenol secretion by Dai 8245 attains a maximum of 12.107 ± 0.056 μg/mL on day 10 and subsequently maintains a steady trend. The polyphenol content of Dai 9061 increases over the whole cultivation process, reaching a maximum of 12.238 ± 0.054 μg/mL on day 14. *Sanghuangporus weigelae* Dai 15768 displays higher polyphenol levels on days 8 and 14, but it remains constant at other periods, achieving a maximum of 6.964 ± 0.123 μg/mL (Figure 3G). As depicted in Figure 3H, the polyphenol yield of *S. zonatus* Dai 10841 continues to increase during the whole cultivation process, with the highest level of 10.567 ± 0.175 μg/mL recorded on day 14.

The rank orders for the top five strains with the highest polyphenol contents were as follows: *S. vaninii* Dai 8236 (12.758 ± 0.133 μg/mL) > *S. vaninii* Dai 9061 (12.238 ± 0.054 μg/mL) > *S. vaninii* Dai 8245 (12.107 ± 0.056 μg/mL) > *S. zonatus* Dai 10841 (10.567 ± 0.175 μg/mL) > *S. lonicericola* Dai 17304 (9.663 ± 0.114 μg/mL). In addition to a few strains that continuously secreted polyphenols, the yields of most strains varied within a certain range, which was beneficial for screening those with higher polyphenol contents. In this study, the polyphenol contents of the three wild *S. vaninii* strains were significantly higher than those of other strains examined, similar to the results obtained by Song et al. [30]. They confirmed that the polyphenol secretion by *S. vaninii* was almost consistently higher than those by *S. baumii* and *S. sanghuang* during the whole cultivation period, with a maximum of 0.416 μg/mL. Further studies are required to validate whether *S. vaninii* species of the genus *Sanghuangporus* potentially possess a stronger ability to produce polyphenols and the underlying mechanisms.

### 3.5. Flavonoid Content

**Figure 4:**
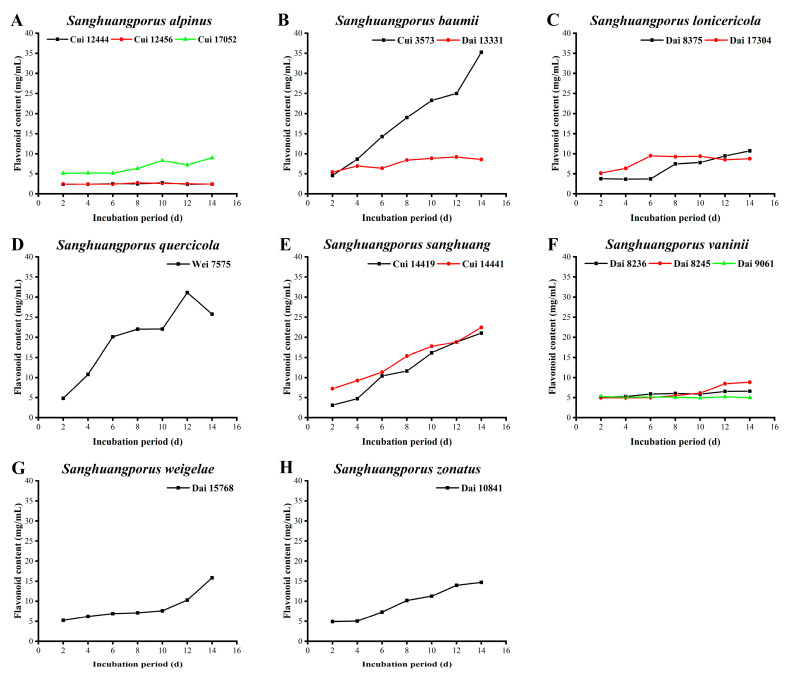
Variations in flavonoid contents during submerged fermentation of 15 wild strains from 8 species of *Sanghuangporus*. (**A**) *Sanghuangporus alpinus* Cui 12444, Cui 12456, and Cui 17052. (**B**) *Sanghuangporus baumii* Cui 3573 and Dai 13331. (**C**) *Sanghuangporus lonicericola* Dai 8375 and Dai 17304. (**D**) *Sanghuangporus quercicola* Wei 7575. (**E**) *Sanghuangporus sanghuang* Cui 14419 and Cui 14441. (**F**) *Sanghuangporus vaninii* Dai 8236, Dai 8245, and Dai 9061. (**G**) *Sanghuangporus weigelae* Dai 15768. (**H**) *Sanghuangporus zonatus* Dai 10841.

Flavonoids are secondary metabolites widely distributed in plants and higher fungi. Owing to the existence of 2-phenyl chromogenic ketones, several benzene rings, and phenolic hydroxyl groups in their structure, flavonoids can also act as electron donors to perform a large number of medicinal functions [53,54,55]. The flavonoid yields of the 15 wild strains from 8 species of *Sanghuangporus* determined in this study are displayed in Figure 4 and Supplementary Table S1.

As illustrated in Figure 4A, the three wild *S. alpinus* strains possess the lowest flavonoid levels, in particular Cui 12444 and Cui 12456. For Cui 17052, a slight elevation in flavonoid yield from 5.120 ± 0.000 to 8.996 ± 0.014 mg/mL was observed. The flavonoid yields of the two wild *S. baumii* strains vary over a wide range (Figure 4B). Cui 3573 fluctuates significantly from 4.578 ± 0.009 to 35.261 ± 0.118 mg/mL, whereas Dai 13331 exhibits a slight increase in flavonoids between 5.422 ± 0.001 and 9.197 ± 0.015 mg/mL. The flavonoid contents of the two wild *S. lonicericola* strains are maintained at low levels during the whole cultivation period (Figure 4C). The flavonoid content of Dai 8375 is lower than that of Dai 17304 in the first 10 days, but higher from days 11 to 14, with a maximum of 10.723 ± 0.022 mg/mL on day 14. These results are markedly higher than that of *S. lonicericola* obtained by Wang et al. [29], with a maximum of 4.45 mg/mL on day 10. The flavonoid content of *S. quercicola* Wei 7575 displays a downward trend after showing a continuous increase from 4.819 ± 0.005 to 31.124 ± 0.113 mg/mL in the first 12 days. Until day 14, its flavonoid content remains at 25.763 ± 0.044 mg/mL (Figure 4D). As shown in Figure 4E, flavonoid secretion by the two wild *S. sanghuang* strains Cui 14419 (21.044 ± 0.333 mg/mL) and Cui 14441 (22.430 ± 0.044 mg/mL) increases during the whole cultivation process, with the highest levels on day 14. Notably, the capacity of Cui 14441 to synthesize flavonoids is greater than that of Cui 14419 throughout the 14-day incubation period. No obvious variations were observed in the flavonoid yields of Dai 8236 and Dai 9061 (Figure 4F). The flavonoid content of Dai 8245 is synchronized with Dai 8236 and Dai 9061 during the early and middle stages, but it increases from days 10 to 14, with a maximum level of 8.855 ± 0.009 mg/mL. The flavonoid contents of *S. weigelae* Dai 15768 and *S. zonatus* Dai 10841 continue to increase during the 14-day cultivation period. The highest levels were calculated as 15.823 ± 0.100 and 14.699 ± 0.038 mg/mL, respectively (Figure 4G,H).

Among the strains examined, *S. baumii* Cui 3573 displayed the highest ability to synthesize flavonoids (35.261 ± 0.118 mg/mL), followed by *S. quercicola* Wei 7575 (31.124 ± 0.113 mg/mL), *S. sanghuang* Cui 14441 (22.430 ± 0.044 mg/mL), and *S. sanghuang* Cui 14419 (21.044 ± 0.333 mg/mL). Flavonoid is one of the characteristic bioactive components of *Sanghuangporus* and serves as an important indicator of bioactivity [56]. The flavonoid yields of the strains examined either remained relatively stable or increased with the growth of mycelial biomass. Additionally, the flavonoid contents derived from different strains varied significantly (such as *S. baumii* Cui 3573 and Dai 13331), which could be attributed to their different habitats, contributing to the selection and utilization of strains with higher flavonoid contents.

### 3.6. Triterpenoid Content

**Figure 5:**
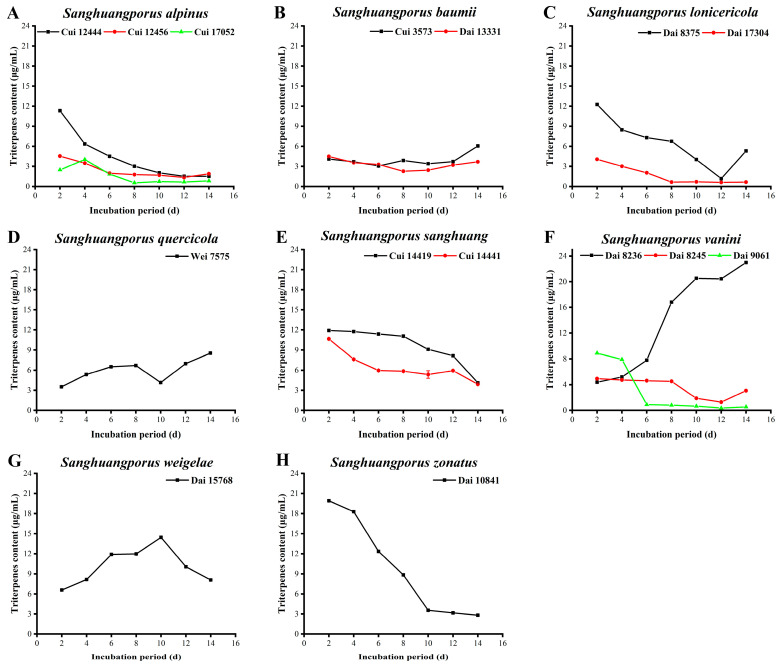
Variations in triterpenoid contents during submerged fermentation of 15 wild strains from 8 species of *Sanghuangporus*. (**A**) *Sanghuangporus alpinus* Cui 12444, Cui 12456, and Cui 17052. (**B**) *Sanghuangporus baumii* Cui 3573 and Dai 13331. (**C**) *Sanghuangporus lonicericola* Dai 8375 and Dai 17304. (**D**) *Sanghuangporus quercicola* Wei 7575. (**E**) *Sanghuangporus sanghuang* Cui 14419 and Cui 14441. (**F**) *Sanghuangporus vaninii* Dai 8236, Dai 8245, and Dai 9061. (**G**) *Sanghuangporus weigelae* Dai 15768. (**H**) *Sanghuangporus zonatus* Dai 10841.

Triterpenoids are another class of biologically active compounds with diverse pharmacological properties, such as anti-tumor, anti-inflammatory, and anti-aging activities [57,58,59]. The triterpenoid yields of the 15 wild strains from 8 species of *Sanghuangporus* are presented in Figure 5 and Supplementary Table S1.

Apart from an increase in triterpenoids in Cui 17052 on day 4, the contents of the three wild *S. alpinus* strains decrease overall (Figure 5A). The maximum triterpenoid levels secreted by Cui 12444, Cui 12456, and Cui 17052 were estimated as 11.332 ± 0.093, 4.547 ± 0.056, and 4.031 ± 0.083 μg/mL on days 2, 2, and 4, respectively. As shown in Figure 5B, the triterpenoid contents of the two wild *S. baumii* strains show a slight increase after decreasing in the early and middle phases, while that of Dai 13331 does not return to the initial level. The maximum triterpenoid yields of Cui 3573 and Dai 13331 are 6.045 ± 0.073 and 4.487 ± 0.049 μg/mL obtained on days 14 and 2, respectively. In Figure 5C, the triterpenoid yield of Dai 8375 decreases from 12.255 ± 0.083 μg/mL to a minimum of 1.174 ± 0.013 μg/mL on day 12 and recovers slightly in the final two days. The triterpenoid content of Dai 17304 decreases from 4.070 ± 0.048 to 0.658 ± 0.005 μg/mL between days 2 to 8, and it persistently stays low over the last few days. The triterpenoid level of Dai 8375 is markedly higher than that of Dai 17304 at all times. Triterpenoid secretion by *S. quercicola* Wei 7575 increases steadily in the first eight days, but it shows a decrease between days 8 to 10. Thereafter, the level starts to increase again, with the maximum value of 8.535 ± 0.037 μg/mL observed on day 14 (Figure 5D). Similarly, the triterpenoid contents of the two wild *S. sanghuang* strains Cui 14419 and Cui 14441 continuously decrease (from 11.908 ± 0.071 to 4.120 ± 0.049 μg/mL and from 10.658 ± 0.055 to 3.902 ± 0.56 μg/mL, respectively) during the whole cultivation process (Figure 5E). Cui 14419 consistently displays a stronger ability to synthesize triterpenoids than Cui 14441. The triterpenoid contents of the three wild *S. vaninii* strains are depicted in Figure 5F. In contrast to other strains, the ability of Dai 8236 to secrete triterpenoids increases during the cultivation process. Elevated levels were observed from days 6 to 8, and a maximum value of 22.979 ± 0.075 μg/mL was recorded on day 14. In contrast, the triterpenoid contents of Dai 8245 and Dai 9061 show a decreasing trend, with maximum values of 4.934 ± 0.023 and 8.882 ± 0.013 μg/mL on day 2, respectively. The triterpenoid yields of *S. weigelae* Dai 15768 increase steadily from 6.580 ± 0.031 μg/mL on day 2 to 14.438 ± 0.177 μg/mL on day 10 and subsequently decrease in the next four days (Figure 5G). As indicated in Figure 5H, the triterpenoid content of *S. zonatus* Dai 10841 is markedly reduced from 19.914 ± 0.108 to 2.815 ± 0.012 μg/mL during the process of cultivation.

The top five strains with higher triterpenoid contents were ranked in the following order: *S. vaninii* Dai 8236 (22.979 ± 0.075 μg/mL) > *S. zonatus* Dai 10841 (19.914 ± 0.108 μg/mL) > *S. weigelae* Dai 15768 (14.438 ± 0.177 μg/mL) > *S. lonicericola* Dai 8375 (12.255 ± 0.083 μg/mL) > *S. sanghuang* Cui 14419 (11.908 ± 0.071 μg/mL). Triterpenoid synthesis by most strains exhibited a decreasing trend, which could be credited to the preference of *Sanghuangporus* species to secrete polysaccharides, flavonoids, and polyphenols to promote resistance against environmental stress. Moreover, the culture conditions used may not have been suitable for the accumulation of triterpenoids.

### 3.7. Ascorbic Acid Content

**Figure 6:**
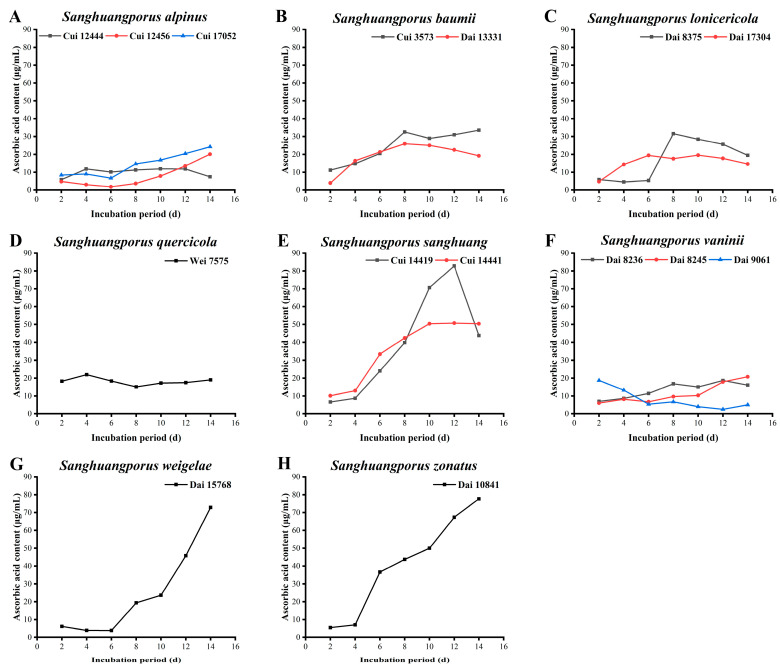
Variations in ascorbic acid contents during submerged fermentation of 15 wild strains from 8 species of *Sanghuangporus*. (**A**) *Sanghuangporus alpinus* Cui 12444, Cui 12456, and Cui 17052. (**B**) *Sanghuangporus baumii* Cui 3573 and Dai 13331. (**C**) *Sanghuangporus lonicericola* Dai 8375 and Dai 17304. (**D**) *Sanghuangporus quercicola* Wei 7575. (**E**) *Sanghuangporus sanghuang* Cui 14419 and Cui 14441. (**F**) *Sanghuangporus vaninii* Dai 8236, Dai 8245, and Dai 9061. (**G**) *Sanghuangporus weigelae* Dai 15768. (**H**) *Sanghuangporus zonatus* Dai 10841.

AAs, also termed vitamin C, are acidic polyhydroxy compounds containing six carbon atoms and are often used as standards for determination of antioxidant activities, owing to their strong reducing power and ability to scavenge endogenous oxygen free radicals [60,61]. The data on the AA contents of the 15 wild strains from 8 species of *Sanghuangporus* are listed in Figure 6 and Supplementary Table S1.

The abilities of the three wild *S. alpinus* strains to synthesize AAs are shown in Figure 6A. AAs secreted by Cui 12444 initially increases from 5.803 ± 0.006 to 11.831 ± 0.011 μg/mL and subsequently stabilizes from days 4 to 12. A slight decrease was observed in the last two days. The AA yields of Cui 12456 and Cui 17052 show a downward trend in the early stages, decreasing to a minimum level on day 6. Subsequently, the AA levels increase steadily, with the highest levels of 20.113 ± 0.051 and 24.282 ± 0.031 μg/mL, respectively, measured on day 14 for both strains. The AA contents of the two wild *S. baumii* strains are presented in Figure 6B. The content of Dai 13331 increases to a maximum level of 25.971 ± 0.022 μg/mL on day 8, followed by a downward trend. The ability of Cui 3573 to yield AAs maintains an upward trend overall, and a maximum of 33.577 ± 0.008 μg/mL was obtained on day 14. The AA secretion by the two wild *S. lonicericola* strains is shown in Figure 6C. The AA content of Dai 8375 exhibits an unremarkable change in the early stages, followed by a subsequent increase on day 6, attaining a maximum level of 31.606 ± 0.077 μg/mL on day 8. The AA-forming ability of Dai 17304 shows an upward trend in the early stages and reaches a maximum of 19.549 ± 0.004 μg/mL on day 10. The AA-synthesizing ability of Dai 17304 is stronger than that of Dai 8375 in the early stages, but it reduces from day 7 onwards. No significant changes were observed in the AA content of *S. quercicola* Wei 7575. The maximum value was measured as 21.972 ± 0.011 μg/mL on day 4 (Figure 6D). However, the results are significantly higher than those of *S. lonicericola* (46.58 μmol/L) and *S. quercicola* (77.11 μmol/L) detected by Wang et al. [29], possibly owing to the differences in incubation, testing, and operational conditions. The AA contents of the two wild *S. sanghuang* strains significantly increase (Figure 6E). AAs derived from Cui 14419 increase from 6.648 ± 0.006 μg/mL on day 2 to a maximum of 82.817 ± 0.072 μg/mL on day 12, and then the level subsequently decreases to 43.775 ± 0.016 μg/mL on day 14. The ability of Cui 14441 to yield AAs increases from days 2 to 10 and then maintains a steady trend, with the highest level determined as 50.704 ± 0.017 μg/mL on day 12. The AA secretion abilities of the three wild *S. vaninii* strains are presented in Figure 6F. The AA contents of Dai 8245 and Dai 8236 continue to increase during the cultivation process, with estimated maximum levels of 20.789 ± 0.046 and 18.648 ± 0.011 μg/mL, respectively. In contrast, the AA content of Dai 9061 presents a downward trend, with the highest level of 18.704 ± 0.020 μg/mL on day 2. A moderate decline in the AA content of *S. weigelae* Dai 15768 (from 6.197 ± 0.020 to 3.887 ± 0.003 μg/mL) is detected in the early stages. Thereafter, the AA content increases, attaining a maximum of 72.845 ± 0.177 μg/mL on day 14 (Figure 6G). The AA level of *S. zonatus* Dai 10841 increases slowly in the first four days, followed by a significant escalation to day 14, with the maximum level measured as 77.690 ± 0.172 μg/mL (Figure 6H).

*S. sanghuang* Cui 14419 (82.817 ± 0.072 μg/mL) exerted the highest functionality in terms of AA yield, followed by *S. zonatus* Dai 10841 (77.690 ± 0.172 μg/mL), *S. weigelae* Dai 15768 (72.845 ± 0.177 μg/mL), and *S. sanghuang* Cui 14441 (50.704 ± 0.017 μg/mL). Moreover, strains with stronger AA-secreting capacities showed a similar trend. Specifically, AA contents increased with the growth of mycelial biomass. Identification of the bioactive ingredients of natural food and health care products is essential to meet the demands for a balanced diet and healthy lifestyle. These findings demonstrate that the large quantities of AAs accumulated by *Sanghuangporus* strains at the later stages of submerged fermentation support the utility of this component in the health benefits of medicinal fungi.

### 3.8. Hydroxyl Radical Scavenging Activity

**Figure 7:**
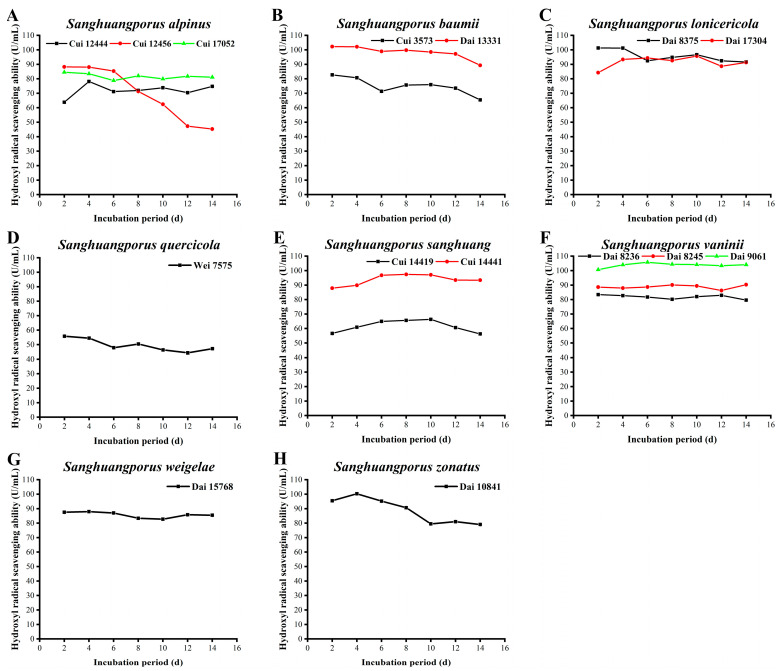
Variations in hydroxyl radical scavenging activities during submerged fermentation of 15 wild strains from 8 species of *Sanghuangporus*. (**A**) *Sanghuangporus alpinus* Cui 12444, Cui 12456, and Cui 17052. (**B**) *Sanghuangporus baumii* Cui 3573 and Dai 13331. (**C**) *Sanghuangporus lonicericola* Dai 8375 and Dai 17304. (**D**) *Sanghuangporus quercicola* Wei 7575. (**E**) *Sanghuangporus sanghuang* Cui 14419 and Cui 14441. (**F**) *Sanghuangporus vaninii* Dai 8236, Dai 8245, and Dai 9061. (**G**) *Sanghuangporus weigelae* Dai 15768. (**H**) *Sanghuangporus zonatus* Dai 10841.

Reactive oxygen species (ROS) are a class of chemically active compounds that are molecularly composed of oxygen. ROS account for 95% total radicals in organisms and include hydroxyl radicals, superoxide radicals, peroxide hydrogen, singlet oxygen, and triplet oxygen [62,63]. Under normal physiological conditions, ROS are in a dynamic equilibrium between production and removal by the antioxidant systems of organisms and known to be capable of sterilization, detoxification, cellular signal transduction, and stimulation of growth, division, and apoptosis. Once equilibrium is disrupted through physical or chemical factors, excess ROS can trigger oxidative damage and destroy lipids, DNA, proteins, and polysaccharides, thus posing a significant threat to organisms [64]. Hydroxyl radicals, regarded as the most active ROS species that pose a considerable health risk, are usually generated by the Fenton reaction and iron-catalyzed Haber–Weiss reaction [65]. Therefore, determination of the hydroxyl radical scavenging activities of the 15 wild strains from 8 species of *Sanghuangporus* may be of great significance in the preparation of therapeutic drugs, vaccines, and functional foods [66]. As shown in Figure 7 and Supplementary Table S1, the hydroxyl radical scavenging activities of the majority of strains in this study are relatively stable during the culture process.

The hydroxyl radical scavenging activities of the three wild *S. alpinus* strains are illustrated in Figure 7A. The activity of Cui 12444 increases to 78.160 ± 0.051 U/mL on day 4 and then holds steady after a slight drop. The capacity of Cui 12456 to clear up hydroxyl radicals is highest (88.240 ± 0.005 U/mL) on day 2 and gradually declines from day 6. The level of activity remains at 45.304 ± 0.204 U/mL until day 14. The activity of Cui 17052 is maintained between 78.825 ± 0.013 and 84.531 ± 0.008 U/mL during the 14-day incubation period. The scavenging activity of *S. baumii* Dai 13331 is markedly higher than that of *S. baumii* Cui 3573. Both show a moderate decrease with increasing incubation time, with maximum values of 102.267 ± 0.005 (Dai 13331) and 82.724 ± 0.014 U/mL (Cui 3573) on day 2, respectively (Figure 7B). As displayed in Figure 7C, the hydroxyl radical scavenging activities of the two wild *S. lonicericola* strains are different in the early stages. From day 6, their activities stabilize and basically coincide, with the highest levels determined as 101.316 ± 0.010 (Dai 8375) and 95.753 ± 0.011 U/mL (Dai 17304), respectively. The ability of *S. quercicola* Wei 7575 to remove hydroxyl radicals is weak and decreases slightly during the cultivation process, from a maximum of 55.812 ± 0.017 U/mL on day 2 to a minimum of 44.353 ± 0.017 U/mL on day 12 (Figure 7D). The scavenging activities of the two wild *S. sanghuang* strains against hydroxyl radicals are relatively steady. Overall, the ability of Cui 14441 is significantly stronger than that of Cui 14419 (Figure 7E). Cui 14419 shows stable activity (between 56.288 ± 0.007 and 66.320 ± 0.067 U/mL) similar to Cui 14441 (between 87.860 ± 0.028 and 97.417 ± 0.006 U/mL). In both cases, maximum activities are achieved during the middle stages of the 14-day incubation period. Among the three wild *S. vaninii* strains (Figure 7F), the hydroxyl radical scavenging activity of Dai 9061 is the strongest, followed by Dai 8245 and Dai 8236. The maximum activities are 105.752 ± 0.022, 90.285 ± 0.017, and 83.438 ± 0.008 U/mL, as observed on days 6, 14, and 2, respectively. The hydroxyl radical scavenging activity of *S. weigelae* Dai 15768 remains basically unchanged during the cultivation process, with a maximum of 87.955 ± 0.005 U/mL on day 4 (Figure 7G). The scavenging activity of *S. zonatus* Dai 10841 is maximum (100.317 ± 0.009 U/mL) on day 4, followed by a decline, and it remains stable after day 10. Even on day 14, its activity remains over 79 U/mL (Figure 7H).

In the present study, the top four strains with hydroxyl radical scavenging activities (>100 U/mL) were as follows: *S. vaninii* Dai 9061 (105.752 ± 0.022 U/mL), *S. baumii* Dai 13331 (102.267 ± 0.005 U/mL), *S. lonicericola* Dai 8375 (101.316 ± 0.010 U/mL), and *S. zonatus* Dai 10841 (100.317 ± 0.009 U/mL). The hydroxyl radical scavenging activities of wild *Sanghuangporus* strains appear strong and stable, highlighting their potential applicability in natural resource-based nutraceuticals, pharmaceuticals, and cosmeceuticals. This conclusion is also supported by Song et al. [30], who determined that the maximum scavenging activities of *S. baumii*, *S. sanghuang*, and *S. vaninii* against hydroxyl radicals were 90.08%, 94.06%, and 87.61%, respectively.

### 3.9. Superoxide Radical Scavenging Activity

**Figure 8:**
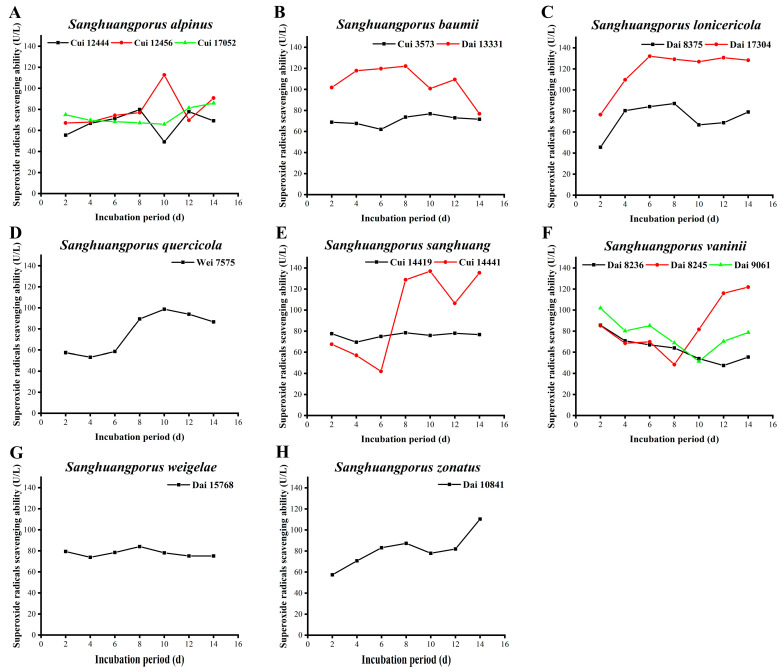
Variations in superoxide radical scavenging activities during submerged fermentation of 15 wild strains from 8 species of *Sanghuangporus.* (**A**) *Sanghuangporus alpinus* Cui 12444, Cui 12456, and Cui 17052. (**B**) *Sanghuangporus baumii* Cui 3573 and Dai 13331. (**C**) *Sanghuangporus lonicericola* Dai 8375 and Dai 17304. (**D**) *Sanghuangporus quercicola* Wei 7575. (**E**) *Sanghuangporus sanghuang* Cui 14419 and Cui 14441. (**F**) *Sanghuangporus vaninii* Dai 8236, Dai 8245, and Dai 9061. (**G**) *Sanghuangporus weigelae* Dai 15768. (**H**) *Sanghuangporus zonatus* Dai 10841.

As a precursor of other ROS, such as singlet oxygen, hydrogen peroxide, and hydroxyl radical, superoxide radicals possess strong oxidizing power and play an important role in metabolism [67]. Superoxide has unpaired electrons and is usually generated via autoxidation of pyrogallol. When autoxidation reaction occurs for 30–40 s, accumulating concentrations of intermediates display a linear (4–5 min) relationship with the reaction time. The removal rate of superoxide radicals could be determined by calculating the autoxidation rate, hence reflecting the antioxidant activities of natural products [68]. The abilities of the 15 wild strains from 8 species of *Sanghuangporus* to remove superoxide radicals were investigated to evaluate their antioxidant activity. The results are displayed in Figure 8 and Supplementary Table S1.

The superoxide radical scavenging activities of the three wild *S. alpinus* strains are illustrated in Figure 8A. Apart from day 10, no significant variations among the three strains were observed. Their maximal removal abilities were detected as 112.674 ± 0.057 (Cui 12456), 85.930 ± 0.015 (Cui 17052), and 79.884 ± 0.030 U/L (Cui 12444) on days 10, 14, and 8, respectively. The superoxide radical scavenging abilities of the two wild *S. baumii* strains are shown in Figure 8B. In the early phases of the 14-day cultivation period, the scavenging activity of Dai 13331 is markedly higher than that of Cui 3573. With an extension of the incubation period, the antioxidant activity of Dai 13331 declines, whereas that of Cui 3573 slightly increases. Until day 14, the scavenging activities of the secreted compounds are almost the same. The maximum activities of Dai 13331 and Cui 3573 were estimated as 122.093 ± 0.008 and 76.860 ± 0.050 U/L on days 8 and 10, respectively. In terms of mycelial biomass, degeneration of the strain may be responsible for the decline in the scavenging capacity of Dai 13331 at the late stages of incubation. The superoxide radical scavenging activities of the two wild *S. lonicericola* strains rapidly increase, followed by a decrease, and these activities subsequently stabilize (Figure 8C). However, the scavenging capacity of Dai 17304 is stronger than that of Dai 8375. Their maximum activities were determined as 132.093 ± 0.016 (Dai 17304) and 87.093 ± 0.014 U/L (Dai 8375) on days 6 and 8, respectively. In the early stages, the activity of *S. quercicola* Wei 7575 in quenching superoxide radicals is relatively stable owing to the adaptation stages. From days 6 to 10, more free radicals are produced by the ever-aging strain, resulting in a gradual increase in scavenging ability. A maximum activity of 98.721 ± 0.024 U/L was measured on day 10 (Figure 8D). Ultimately, the scavenging ability decreases with severe aging and even autolysis of the strain. Unlike this study, Wang et al. [29] observed that the quenching activities of *S. lonicericola* and *S. quercicola* showed a continuing downward trend, with maximum values of 57.82% and 65.48% on days 4 and 6, respectively. As displayed in Figure 8E, the removal capacity of *S. sanghuang* Cui 14419 against superoxide radicals is basically stable (at ~70 U/L) during the incubation process, while the activity of *S. sanghuang* Cui 14441 fluctuates dramatically. During the early phases of cultivation, the scavenging activity of Cui 14441 is lower than that of Cui 14419, which could be due to the adaptation period. From days 6 to 8, the capacity of Cui 14441 increases and surpasses that of Cui 14419. Cui 14441 attains a maximum scavenging activity of 136.977 ± 0.006 U/L on day 10. The superoxide radical scavenging activities of the three wild *S. vaninii* strains present a trend showing an initial decrease followed by an increase (Figure 8F). This could be attributed to the fact that in the early stages of incubation, the strains grew vigorously, and a large number of superoxide radicals were transformed into other ROS, leading to a decrease in scavenging activity. With continuing culture time, the ever-aging strains and the excessive production of free radicals led to a rebound in scavenging ability. The maximum quenching activities were estimated as 85.698 ± 0.027 (Dai 8236), 121.860 ± 0.016 (Dai 8245), and 101.860 ± 0.041 U/L (Dai 9061), observed on days 2, 14, and 2, respectively. The scavenging activity of *S. weigelae* Dai 15768 is stable during the 14-day incubation process, maintaining between 73.837 ± 0.066 and 84.070 ± 0.022 U/L (Figure 8G). The superoxide radical scavenging activity of *S. zonatus* Dai 10841 increases from 57.442 ± 0.069 to 87.326 ± 0.093 U/L between days 2 and 8, and it declines from days 8 to 10. The scavenging ability is subsequently restored, attaining a maximum level of 110.349 ± 0.047 U/L on day 14 (Figure 8H). These variations could be explained by the fact that the metabolism of the strain slows down at the late stages of growth, resulting in a weaker quenching capacity of superoxide radicals.

The top five strains with the strongest superoxide radical scavenging activities were ranked as follows: *S. sanghuang* Cui 14441 (136.977 ± 0.006 U/L) > *S. lonicericola* Dai 17304 (132.093 ± 0.016 U/L) > *S. baumii* Dai 13331 (122.093 ± 0.008 U/L) > *S. vaninii* Dai 8245 (121.860 ± 0.016 U/L) > *S. alpinus* Cui 12456 (112.674 ± 0.057 U/L). Overall, the capacities of other strains to quench superoxide radicals were relatively weak. Moreover, significant differences in superoxide radical scavenging capacities were detected among the same species of the genus *Sanghuangporus*, which could probably be attributed to the various precursors of ROS, antioxidant activities of strains, and distinctive microenvironments [12].

### 3.10. DPPH Radical Scavenging Activity

**Figure 9:**
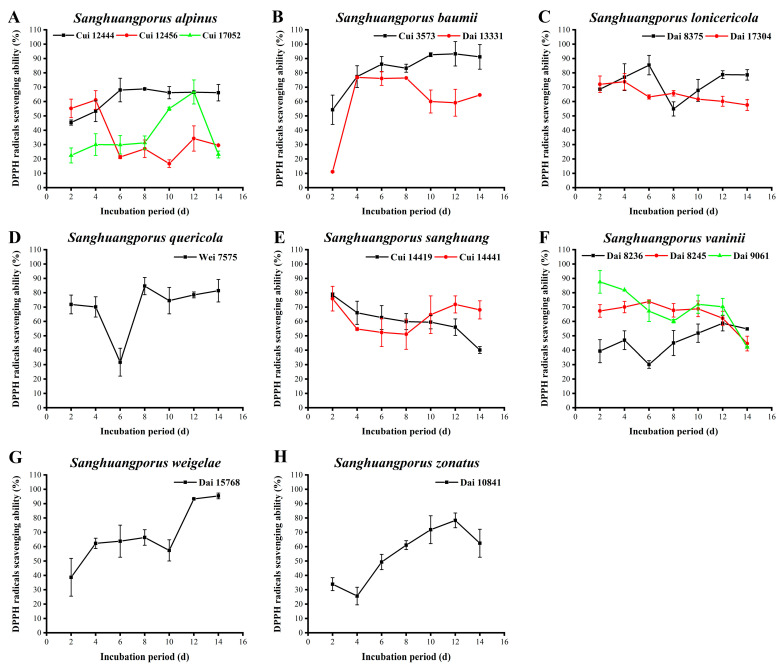
Variations in DPPH radical scavenging activities during submerged fermentation of 15 wild strains from 8 species of *Sanghuangporus*. (**A**) *Sanghuangporus alpinus* Cui 12444, Cui 12456, and Cui 17052. (**B**) *Sanghuangporus baumii* Cui 3573 and Dai 13331. (**C**) *Sanghuangporus lonicericola* Dai 8375 and Dai 17304. (**D**) *Sanghuangporus quercicola* Wei 7575. (**E**) *Sanghuangporus sanghuang* Cui 14419 and Cui 14441. (**F**) *Sanghuangporus vaninii* Dai 8236, Dai 8245, and Dai 9061. (**G**) *Sanghuangporus weigelae* Dai 15768. (**H**) *Sanghuangporus zonatus* Dai 10841.

DPPH is a stable radical, and its alcohol solution is purple with a strong absorption peak at 517 nm. Upon pairing of the single electron of DPPH, the color of the alcohol solution changes from purple to yellow. The degree of color fading is quantitatively related to the number of accepted electrons. Accordingly, DPPH radicals can be used as a characteristic indicator for the rapid detection of antioxidant activities of natural products via this colorimetric method [69]. The scavenging activities against DPPH radicals of the 15 wild strains from 8 species of *Sanghuangporus* were evaluated in this study. The results are shown in Figure 9 and Supplementary Table S1.

The scavenging activities of the three wild *S. alpinus* strains are shown in Figure 9A. The activity of Cui 12444 increases rapidly between days 2 and 6, and it remains constant over the last eight days, with the highest activity of 68.832 ± 0.399% on day 8. The scavenging activity of Cui 17052 exhibits a small fluctuation in the early stages and increases from day 8 of the incubation period. Finally, the activity returns to the original level on day 14, showing a maximum of 66.729 ± 8.368% on day 12. The activity of Cui 12456 decreases to 21.343 ± 1.071% on day 6, which is lower than those of Cui 12444 and Cui 17052, and then fluctuates within a certain range (from 16.761 ± 2.658% to 34.275 ± 8.789%) between days 6 and 14. The lower polyphenol, flavonoid, and AA yields of Cui 12456 may lead to the lower DPPH radical scavenging activity relative to Cui 12444 and Cui 17052. Figure 9B depicts the DPPH radical scavenging activities of the two wild *S. baumii* strains. The ability of Cui 3573 to scavenge DPPH radicals exerts a relatively steady trend after an initial increase. The maximum level was estimated as 93.296 ± 8.501% on day 12. The activity of Dai 13331 increases from 11.111 ± 0.533% on day 2 to 76.836 ± 0.799% on day 4, remaining stable over the next four days. The decline in scavenging activity from days 8 to 10 could probably be attributed to the progressive degeneration of the strain. Notably, the scavenging activity of Cui 3573 is higher than that of Dai 13331, especially at the later stages, possibly as a result of higher growth activity and secretion of more active ingredients. As presented in Figure 9C, the activity of *S. lonicericola* Dai 17304 in quenching DPPH radicals decreases slowly over the incubation period, with higher values of 72.065 ± 5.766% and 73.823 ± 5.709% on days 2 and 4, which may be ascribed to the extension of culture time. This strain exhibits a delay in growth, metabolism, and antioxidant capacity. The activity of Dai 8375 increases during the initial cultural phases, attaining a maximum of 85.405 ± 6.791% on day 6. In the next two days, the activity decreases to 54.896 ± 4.927% and then increases again on day 8. This finding may be explained by the fact that this strain with degenerative growth and metabolism has to rely on activating its own antioxidant mechanism to resist external damage. The DPPH radical scavenging activity of *S. quercicola* Wei 7575 declines in the early stages, with a low value of 31.701 ± 9.666% on day 6 (Figure 9D). Once the strain has acclimatized to the new microenvironment, the scavenging activity increases to a maximum of 84.652 ± 5.992% on day 8. Afterwards, the activity fluctuates in a certain range (from 74.513±9.219% to 81.450±7.857%) to stimulate the antioxidant mechanism that promotes resistance to oxidative stress. As shown in Figure 9E, the activities of the two wild *S. sanghuang* strains, Cui 14419 and Cui 14441, in eliminating DPPH radicals gradually decrease during the cultivation process, with respective maximum values of 78.531 ± 1.358% and 75.895 ± 8.583% on day 2. The scavenging activity of Cui 14441 is initially lower than that of Cui 14419, but higher at the late stages, possibly owing to the stronger ability of Cui 14441 to secrete polysaccharides and AAs. The DPPH radical scavenging activities of the three wild *S. vaninii* strains are depicted in Figure 9F. The activity of Dai 8236 also exhibits a wave trend, reaching a minimum of 30.131 ± 2.663% on day 6 and a maximum of 58.757 ± 5.327% on day 12. The activity of Dai 8245 displays a moderate increase from the days 2 to 6, and then gradually decreases, attaining a maximum of 73.886 ± 1.196% on day 6. Dai 9061 displays a decrease in activity from 87.571 ± 7.871% to 60.201 ± 1.198% in the first eight days, followed by an increase, and finally, its activity decreases again in the last two days. The scavenging abilities of the three wild *S. vaninii* strains exhibit a trend of decline in the early stages and the last two days. One possible reason for the decrease is non-adaptability of the strains to the environment during the early stages. Another potential factor is decreased growth and metabolism of the strains in the last two days. The DPPH radical scavenging activity of *S. weigelae* Dai 15768 shows a continual increase in the early stages, followed by a downward trend from days 8 to 10 (Figure 9G). In the last four days, the activity increases again, which is in good agreement with the content variations of other active ingredients, such as polysaccharides, polyphenols, flavonoids, and AAs. The strongest activity of Dai 15768 in DPPH radical removal was determined as 95.386 ± 1.997% on day 14. The activity of *S. zonatus* Dai 10841 in scavenging DPPH radicals shows a continuous upward trend after a short decrease, with a maximum of 78.343 ± 5.126% on day 12 (Figure 9H).

*Sanghuangporus* has strong DPPH radical scavenging ability. Among the strains examined, *S. weigelae* Dai 15768 (95.386 ± 1.997%) and *S. baumii* Cui 3573 (93.296 ± 8.501%) were identified as the strongest scavengers against DPPH radicals with >90% elimination rates, followed by *S. vaninii* Dai 9061 (87.571 ± 7.871%), *S. lonicericola* Dai 8375 (85.405 ± 6.791%), and *S. quercicola* Wei 7575 (84.652 ± 5.992%). Surprisingly, the remaining strains could also scavenge > 50% of DPPH radicals in majority of time. The wild species of the genus *Sanghuangporus* are, therefore, capable of serving as excellent DPPH radical scavengers. The results are consistent with a number of previous reports [29,30,56]. For instance, the maximum DPPH radical scavenging activities of *S. baumii* (95.83%), *S. sanghuang* (81.92%), and *S. vaninii* (87.71%) were assayed above 80% by Song et al. [30].

### 3.11. ABTS Radical Scavenging Activity

**Figure 10:**
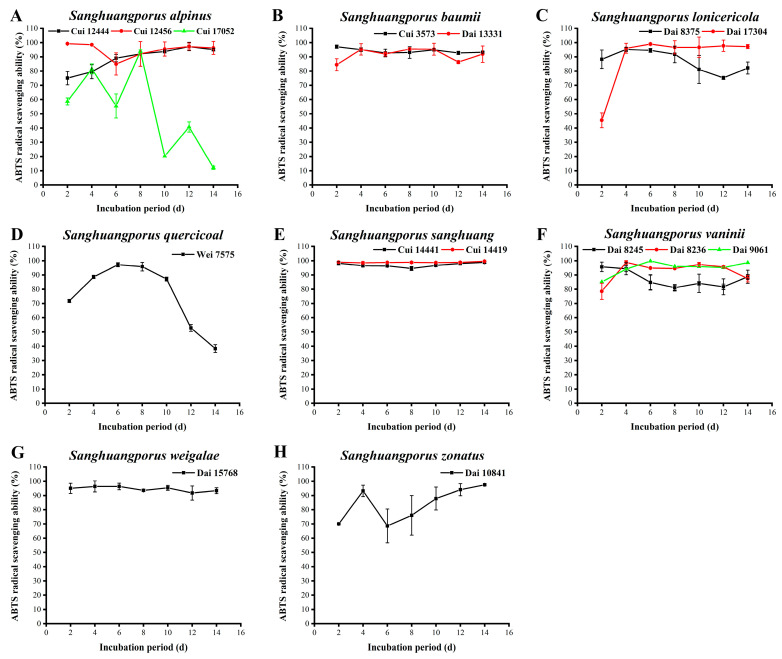
Variations in ABTS radical scavenging activities during submerged fermentation of 15 wild strains from 8 species of *Sanghuangporus*. (**A**) *Sanghuangporus alpinus* Cui 12444, Cui 12456, and Cui 17052. (**B**) *Sanghuangporus baumii* Cui 3573 and Dai 13331. (**C**) *Sanghuangporus lonicericola* Dai 8375 and Dai 17304. (**D**) *Sanghuangporus quercicola* Wei 7575. (**E**) *Sanghuangporus sanghuang* Cui 14419 and Cui 14441. (**F**) *Sanghuangporus vaninii* Dai 8236, Dai 8245, and Dai 9061. (**G**) *Sanghuangporus weigelae* Dai 15768. (**H**) *Sanghuangporus zonatus* Dai 10841.

ABTS, a water-soluble radical, is oxidized by ROS to generate a stable blue-green cationic radical, ABTS^+^, with a characteristic absorption peak at 734 nm. Upon encountering antioxidants, ABTS^+^ is reduced, accompanied by a change in solution color and a decrease in absorbance. Lower absorbance is reflective of stronger ABTS radical scavenging activity of antioxidants [70]. In this study, ABTS radicals were employed to investigate the antioxidant activities of the 15 wild strains from 8 species of *Sanghuangporus* (Figure 10, Supplementary Table S1).

As shown in Figure 10A, the activities of the two wild *S. alpinus* strains Cui 12444 and Cui 12456 in quenching ABTS radicals differ significantly in the first six days, but they are synchronized from day 6. Moreover, the maximum activities of Cui 12444 and Cui 12456 were determined as 97.277 ± 2.952% and 99.257 ± 0.350% on days 12 and 2, respectively. In contrast, the scavenging activity of Cui 17052 fluctuates and declines during the course of incubation. At the late stages of cultivation, the activity of Cui 17052 is significantly lower compared to Cui 12444 and Cui 12456. In view of the finding that Cui 17052 synthesizes higher levels of bioactive ingredients than Cui 12444 and Cui 12456, this discrepancy may be explained by the theory that a coordination of multiple antioxidant mechanisms is required for normal body function. As shown in Figure 10B, other than the relatively low activity of *S. baumii* Dai 13331 in scavenging ABTS radicals on days 2 and 12, the quenching activities of Cui 3573 and Dai 13331 remain strong and stable over the incubation period, which could be attributed to the capacities of the two wild strains to stimulate their antioxidant systems for clearing free radicals that were generated constantly during the culture process. The maximum activities of Cui 3573 and Dai 13331 were assayed as 97.195 ± 1.143% and 95.545 ± 0.857% on days 2 and 8, respectively. The ABTS radical scavenging activities of the two wild *S. lonicericola* strains are displayed in Figure 10C. The activity of Dai 8375 is maximum (95.215 ± 1.512%) on day 4 and declines to a minimum level on day 12. The activity of Dai 17304 increases from 45.462 ± 5.251% to 96.040 ± 3.501% between days 2 and 4. During this time, the strain is in a vigorous growth state, which corresponds to the obvious increase in its antioxidant capacity. Subsequently, the activity of Dai 17304 remains stable, suggesting that this strain initiates a stress mechanism for adaptation to the environment by virtue of maintaining its antioxidant capacity at a certain level. The activity of *S. quercicola* Wei 7575 in quenching ABTS radicals initially increases from 71.782 ± 1.050% to a maximum of 97.195 ± 1.422% on day 6, followed by a gradual decrease (Figure 10D). The data indicate that the ABTS radical scavenging activity of this strain is closely related to its growth status. At the early stages of cultivation, stronger metabolism of the strain is associated with more vigorous growth. With an extension in time and aging of the strain, antioxidant activity gradually decreases. The ABTS radical scavenging activities of the two wild *S. sanghuang* strains are significant and stable during the whole incubation process (Figure 10E). In particular, the activity of Cui 14419 remains at >98%, showing a maximum of 99.670 ± 0.286% on day 14. In Figure 10F, the scavenging activities of the three wild *S. vaninii* strains against ABTS radicals are similarly strong and stable. The activities of Dai 8236 and Dai 9061 increase in a stable manner, with maximum values of 98.845 ± 2.061% and 99.835 ± 0.286% on days 4 and 6, respectively. The activity of Dai 8245 decreases from 95.710 ± 0.756% on day 2 to 84.818 ± 5.381% on day 6, and subsequently remains relatively stable. Although the scavenging activity of Dai 8245 is lower than those of Dai 8236 and Dai 9061, the levels of active ingredients secreted by Dai 8245 are variable, indicating that antioxidant capacity is attributed not to a single function but a series of behaviors of multiple compounds. *Sanghuangporus weigelae* Dai 15768 exerts strong and stable activity during the whole cultivation process, showing a maximum of 96.370 ± 3.845% on day 2 (Figure 10G). *Sanghuangporus zonatus* Dai 10841 harbors a higher scavenging activity against ABTS radicals between days 2 and 4, and its activity decreases thereafter (Figure 10H). To further protect against oxidative damage, Dai 10841 initiates an antioxidant mechanism, which is reflected in the sustainable increase in activity from day 6. The maximum activity level of 97.525 ± 0.561% was reached on day 14.

The majority of *Sanghuangporus* strains employed in the present study exhibited strong ABTS radical scavenging activities. The top five strains were ranked in sequence as follows: *S. vaninii* Dai 9061 (99.835 ± 0.286%), *S. sanghuang* Cui 14419 (99.670 ± 0.286%), *S. alpinus* Cui 12456 (99.257 ± 0.350%), *S. vaninii* Dai 8236 (98.845 ± 2.061%), and *S. sanghuang* Cui 14441 (98.845 ± 0.756%). The scavenging abilities of the *Sanghuangporus* species against ABTS radicals were particularly high compared to other radicals. These findings are consistent with the reports of Zheng et al. [56] and Wang et al. [29] showing that the corresponding scavenging capacities of *S. sanghuang*, *S. lonicericola*, and *S. quercicola* were higher than 90%.

### 3.12. SOD Activity

**Figure 11:**
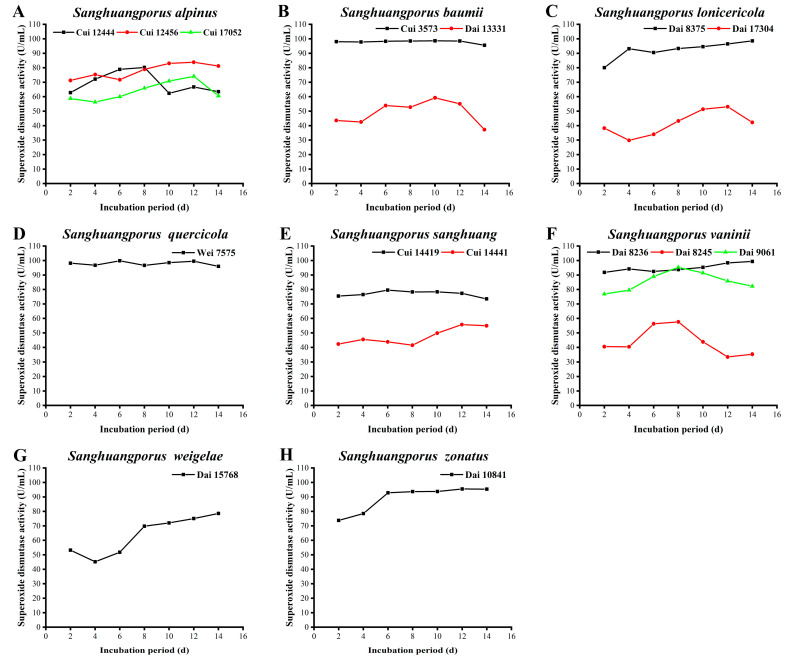
Variations in superoxide dismutase activities during submerged fermentation of 15 wild strains from 8 species of *Sanghuangporus*. (**A**) *Sanghuangporus alpinus* Cui 12444, Cui 12456, and Cui 17052. (**B**) *Sanghuangporus baumii* Cui 3573 and Dai 13331. (**C**) *Sanghuangporus lonicericola* Dai 8375 and Dai 17304. (**D**) *Sanghuangporus quercicola* Wei 7575. (**E**) *Sanghuangporus sanghuang* Cui 14419 and Cui 14441. (**F**) *Sanghuangporus vaninii* Dai 8236, Dai 8245, and Dai 9061. (**G**) *Sanghuangporus weigelae* Dai 15768. (**H**) *Sanghuangporus zonatus* Dai 10841.

SOD composed of protein and metal ions is an important member of the antioxidant metalloenzyme system in living organisms. As an initial barrier against ROS, SOD catalyzes the disproportionation of superoxide radicals into oxygen and hydrogen peroxide. Hydrogen peroxide is further converted into water by catalase and oxidase, thereby scavenging free radicals in cells and maintaining the metabolic balance of the body [71,72]. Additionally, SOD has potential utility in anti-aging, in the improvement of immunity, in the prevention and treatment of various diseases, and as an additive in foods and cosmetics [73,74,75]. The SOD activities of the 15 wild strains from 8 species of *Sanghuangporus* examined in this study are presented in Figure 11 and Supplementary Table S1.

As shown in Figure 11A, the SOD activity of *S. alpinus* Cui 12444 increases slowly in the early periods and reaches a maximum of 80.250 ± 0.010 U/mL on day 8. Thereafter, its activity decreases and then gradually stabilizes. The SOD activities of Cui 12456 and Cui 17052 display simultaneous trends of initial increase followed by decline, with maximal levels of 74.094 ± 0.017 and 83.853 ± 0.007 U/mL, respectively, on day 12. *Sanghuangporus baumii* Cui 3573 possesses a SOD activity > 95 U/mL during the whole cultivation process. The SOD activity of *S. baumii* Dai 13331 is lower than that of Cui 3573 and decreases to 37.272 ± 0.046 U/mL on day 14 (Figure 11B). The reason for this discrepancy may be the differences in the growth status and active ingredient contents of the wild strains. In particular, the mycelial biomass yield of Dai 13331 decreases during the later stages of the 14-day incubation period, and the flavonoid content is markedly lower than that of Cui 3573. *Sanghuangporus lonicericola* Dai 8375 exerts a relatively high SOD activity, increasing from 80.010 ± 0.009 to 98.567 ± 0.013 U/mL between days 2 and 14. The SOD activity of *S. lonicericola* Dai 17304 is predominantly lower than that of Dai 8375, with a minimum of 29.878 ± 0.012 U/mL recorded on day 4. Subsequently, the SOD activity begins to increase again from days 4 to 12, achieving a maximum of 53.075 ± 0.037 U/mL on day 12 (Figure 11C). The activity derived from *S. quercicola* Wei 7575 is considerably high and remains stable throughout the cultivation process, with a maximum of 99.843 ± 0.002 U/mL on day 6 (Figure 11D). The SOD activities of the two wild *S. sanghuang* strains are shown in Figure 11E. Cui 14419 shows a fairly stable activity during the incubation process, which is maintained between 73.531 ± 0.029 and 79.574 ± 0.013 U/mL. The activity of Cui 14441 appears lower than that of Cui 14419, and its maximum was measured as 55.777 ± 0.023 U/mL on day 12. The SOD activities derived from the three wild *S. vaninii* strains are presented in Figure 11F. The activity of Dai 8236 is high and remains relatively stable during the incubation process, reaching a maximum of 99.393 ± 0.008 U/mL on day 14. Dai 9061 displays maximum activity of 95.339 ± 0.019 U/mL on day 8, which subsequently decreases. The ability of Dai 8245 to synthesize SOD increases during the middle stages of cultivation, showing a maximum of 57.654 ± 0.058 U/mL on day 8. Notably, the levels of active ingredients secreted by Dai 8245 are not the lowest, although its SOD activity is obviously lower than those of Dai 8236 and Dai 9061. In view of this finding, it is speculated that the antioxidant activities of these strains depend not only on the varieties and contents of active ingredients, but also on the growth environment and antioxidant mechanisms. As demonstrated in Figure 11G, the capacity of *S. weigelae* Dai 15768 to secrete SOD decreases between days 2 and 4, followed by an increase. At day 14, a maximum SOD activity of 78.598 ± 0.015 U/mL was observed. The SOD activity derived from *S. zonatus* Dai 10841 increases from 73.794 ± 0.012 to 92.862 ± 0.008 U/mL in the early phases and remains constant from days 6 to 14. The highest SOD activity was calculated as 95.414 ± 0.010 U/mL on day 14 (Figure 11H).

The capacities of different *Sanghuangporus* species and strains to yield SOD varied significantly. The rank orders for the top five strains with the higher SOD activities were *S. quercicola* Wei 7575 (99.843 ± 0.002 U/mL) > *S. vaninii* Dai 8236 (99.393 ± 0.008 U/mL) > *S. baumii* Cui 3573 (98.642 ± 0.003 U/mL) > *S. lonicericola* Dai 8375 (98.567 ± 0.013 U/mL) > *S. zonatus* Dai 10841 (95.414 ± 0.010 U/mL). The peak levels were notably higher than those of *S. baumii* (11.43 U/mL), *S. sanghuang* (12.34 U/mL), and *S. vaninii* (12.75 U/mL), which were assayed on day 18 by Song et al. [30]. It has been suggested that it is necessary and important to explore and screen strains with improved activity from different *Sanghuangporus* species and strains.

### 3.13. Ferric Reducing Ability of Plasma

**Figure 12:**
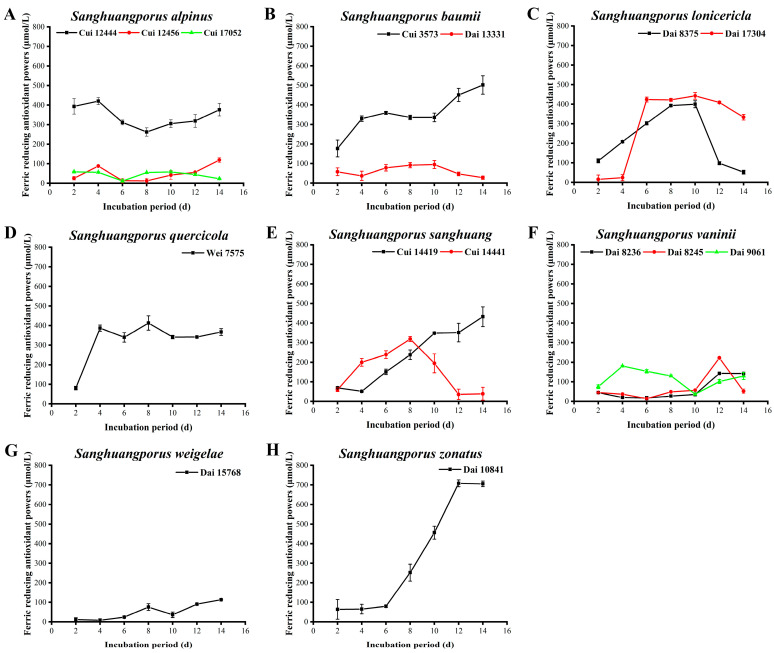
Variations in ferric reducing ability of plasma during submerged fermentation of 15 wild strains from 8 species of *Sanghuangporus*. (**A**) *Sanghuangporus alpinus* Cui 12444, Cui 12456, and Cui 17052. (**B**) *Sanghuangporus baumii* Cui 3573 and Dai 13331. (**C**) *Sanghuangporus lonicericola* Dai 8375 and Dai 17304. (**D**) *Sanghuangporus quercicola* Wei 7575. (**E**) *Sanghuangporus sanghuang* Cui 14419 and Cui 14441. (**F**) *Sanghuangporus vaninii* Dai 8236, Dai 8245, and Dai 9061. (**G**) *Sanghuangporus weigelae* Dai 15768. (**H**) *Sanghuangporus zonatus* Dai 10841.

The antioxidant activities of natural ingredients are mainly dependent on scavenging free radicals, reducing power, inhibiting pro-oxidants (such as chelating transition metals), and blocking of lipid oxidation to generate endogenous antioxidant substances [76]. Determination of a compound’s reducing power provides a useful means to verify whether the compound has excellent electron-supplying capacity. The electrons supplied by compounds with strong reducing power not only reduce oxidizing compounds but also react with free radicals to produce more stable complexes [77]. The FRAP assay, which offers distinct advantages of simple operation and easy standardization, is commonly used to analyze the total antioxidant activities of foods, nutraceuticals, and bioactive ingredients [78]. This assay uses the principle that compounds with antioxidant properties can reduce Fe^3+^ into Fe^2+^, which forms an intense blue complex with TPTZ, exhibiting a characteristic absorption peak at 593 nm [79]. Additionally, the FRAP assay does not reflect the scavenging activity of specific free radicals, but it confirms the total reducing power, which can be applied to comprehensively evaluate the total antioxidant activities of samples [42]. In this study, the FRAP of the 15 wild strains from 8 species of *Sanghuangporus* were determined (Figure 12, Supplementary Table S1).

The FRAP values of the three wild *S. alpinus* strains are shown in Figure 12A. The FRAP value of Cui 12444 is markedly higher than those of Cui 12456 and Cui 17052, with maximum values of 420.149 ± 17.975 (Cui 12444), 119.298 ± 12.638 (Cui 12456), and 68.766 ± 1.107 μM (Cui 17052) on days 4, 14, and 8, respectively. This finding may be indicative of other mechanisms in Cui 12444 contributing to this phenomenon herein that are approximately equal to other indicators. As demonstrated in Figure 12B, the reducing power of Cui 3573 is remarkably higher than that of Dai 13331 and shows an upward trend, reaching a maximum level of 501.993 ± 46.816 μM on day 14. In contrast, the FRAP secreted by *S. baumii* Dai 13331 remains at a low level during the culture process but slightly increases during the middle phases, which could possibly be ascribed to a vigorous growth state of this strain. FRAPs of the two wild *S. lonicericola* strains presented an increasing-stable-decline trend during the incubation process (Figure 12C). The reducing power of Dai 8375 increases to 399.830 ± 17.692 μM on day 10 and subsequently decreases. By day 14, this reducing power is as low as 52.489 ± 9.562 μM. The reducing power of Dai 17304 is considerably weak in the early stages but exceeds that of Dai 8375 from day 6, showing a maximum of 442.489 ± 17.068 μM on day 10. The FRAP of *S. quercicola* Wei 7575 increases from days 2 to 4 and then gradually stabilizes, with a maximum value of 412.809 ± 36.258 μM obtained on day 8 (Figure 12D). As shown in Figure 12E, the reducing power secreted by Cui 14419 increases significantly and reaches a maximum of 432.369 ± 50.401 μM on day 14. Nevertheless, the FRAP of *S. sanghuang* Cui 14441 increases to a maximum level of 318.234 ± 12.879 μM on day 8, followed by a decline. The FRAPs of the three wild *S. vaninii* strains remain low during the cultivation process (Figure 12F). In the first 10 days, the reducing power of Dai 9061 is significantly stronger than those of Dai 8236 and Dai 8245 and attains a maximum of 181.355 ± 3.536 μM on day 4. Over the later periods, the reducing power of Dai 8236 and Dai 8245 increases obviously, achieving maximum values of 143.553 ± 5.357 and 222.170 ± 2.558 μM on day 12, respectively. Although the FRAP of *S. weigelae* Dai 15768 increases at the later stages of culture, the levels remain low, with a maximum of 113.340 ± 0.301 μM obtained on day 14 (Figure 12G). *Sanghuangporus zonatus* Dai 10841 is an interesting strain that shows a distinct pattern from all other strains in the secretion of FRAP (Figure 12H). From days 6 to 12, its reducing power increases apparently from 80.149 ± 5.717 to 708.553 ± 17.903 μM and then stabilizes.

The top five strains with highest FRAPs were ranked as follows: *S. zonatus* Dai 10841 (708.553 ± 17.903 μM) > *S. baumii* Cui 3573 (501.993 ± 46.816 μM) > *S. lonicericola* Dai 17304 (442.489 ± 17.068 μM) > *S. sanghuang* Cui 14419 (432.369 ± 50.401 μM) > *S. alpinus* Cui 12444 (420.149 ± 17.975 μM). Notably, the FRAP of *S. zonatus* Dai 10841 exceeds 700 μM in this study, which is significantly higher than *S. lonicericola*, *S. quercicola*, and *Ganoderma boninense* (400–600 μM) reported by Wang et al. [29] and Yu et al. [80]. In view of this result, further research attention should be devoted to *S. zonatus*.

According to the above data, the most active strains are *S. baumii* Cui 3573, *S. sanghuang* Cui 14419 and Cui 14441, *S. vaninii* Dai 9061, and *S. zonatus* Dai 10841. Since *Sanghuangporus* possesses high quantities of active ingredients with various beneficial biological activities, artificial cultivation of different species and strains of this genus is of great significance for further development and application as therapeutic agents, vaccines, cosmetics, and functional foods. To date, *S. vaninii* and *S. baumii* are among the few *Sanghuangporus* species that have been successfully cultivated. In this study, *S. vaninii* Dai 9061 displayed a higher polyphenol content and stronger ability to scavenge hydroxyl, DPPH, and ABTS radicals. *Sanghuangporus baumii* Cui 3573 yielded greater polysaccharide and flavonoid contents as well as SOD and DPPH radical scavenging activities, conferring its utility as a potential cultivar with strong bioactivity. In addition, the two *S. sanghuang* strains Cui 14419 and Cui 14441, which displayed high levels of polysaccharide, flavonoid, and AA, strong superoxide and ABTS radical scavenging activities, and high FRAP, are superior to other species of *Sanghuangporus* and could provide promising alternatives for further research. High amounts of polyphenol, flavonoid, and AA; stronger radical quenching activity; and higher SOD activity and FRAP were also observed for *S. zonatus* Dai 10841. Limited research attention has focused on *S. zonatus* compared to other well-characterized *Sanghuangporus* species to date. Overall, significant differences in the active ingredients and antioxidant activities among the strains from different species of *Sanghuangporus* were identified in the present study, highlighting the importance of species- and strain-specific selection on the basis of demand.

### 3.14. Correlations between Bioactive Ingredients and Antioxidant Activities

**Figure 13:**
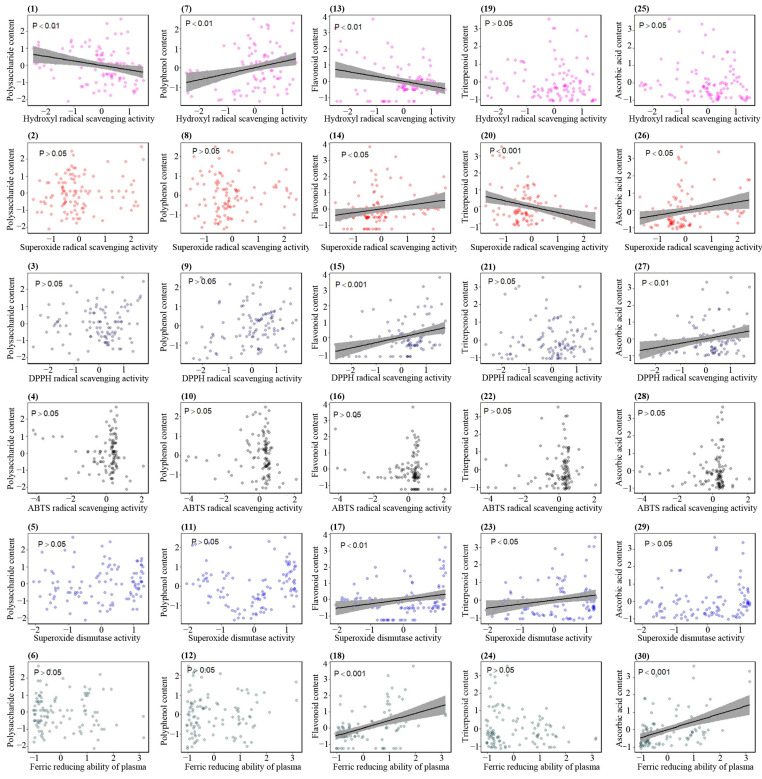
Correlation analysis between bioactive ingredients and antioxidant activities of 15 wild strains from 8 species of *Sanghuangporus*. (**1**) Polysaccharide content and hydroxyl radical scavenging activity. (**2**) Polysaccharide content and superoxide radical scavenging activity. (**3**) Polysaccharide content and DPPH radical scavenging activity. (**4**) Polysaccharide content and ABTS radical scavenging activity. (**5**) Polysaccharide content and superoxide dismutase activity. (**6**) Polysaccharide content and ferric reducing ability of plasma. (**7**) Polyphenol content and hydroxyl radical scavenging activity. (**8**) Polyphenol content and superoxide radical scavenging activity. (**9**) Polyphenol content and DPPH radical scavenging activity. (**10**) Polyphenol content and ABTS radical scavenging activity. (**11**) Polyphenol content and superoxide dismutase activity. (**12**) Polyphenol content and ferric reducing ability of plasma. (**13**) Flavonoid content and hydroxyl radical scavenging activity. (**14**) Flavonoid content and superoxide radical scavenging activity. (**15**) Flavonoid content and DPPH radical scavenging activity. (**16**) Flavonoid content and ABTS radical scavenging activity. (**17**) Flavonoid content and superoxide dismutase activity. (**18**) Flavonoid content and ferric reducing ability of plasma. (**19**) Triterpenoid content and hydroxyl radical scavenging activity. (**20**) Triterpenoid content and superoxide radical scavenging activity. (**21**) Triterpenoid content and DPPH radical scavenging activity. (**22**) Triterpenoid content and ABTS radical scavenging activity. (**23**) Triterpenoid content and superoxide dismutase activity. (**24**) Triterpenoid content and ferric reducing ability of plasma. (**25**) Ascorbic acid content and hydroxyl radical scavenging activity. (**26**) Ascorbic acid content and superoxide radical scavenging activity. (**27**) Ascorbic acid content and DPPH radical scavenging activity. (**28**) Ascorbic acid content and ABTS radical scavenging activity. (**29**) Ascorbic acid content and superoxide dismutase activity. (**30**) Ascorbic acid content and ferric reducing ability of plasma.

To elucidate the relationship between the antioxidant activity of *Sanghuangporus* species and their active ingredients, the correlation between any two indicators was analyzed via a unary linear regression model in the R 4.0.3 software (Figure 13). The results suggest that polysaccharide content is not associated with the antioxidant activity of *Sanghuangporus* and is even negatively correlated with hydroxyl radical scavenging activity, coincident with previous reports [81,82,83]. On the other hand, a highly significant correlation was detected between polyphenol content and hydroxyl radical scavenging activity. Additionally, flavonoid content markedly affects scavenging activities against hydroxyl, superoxide, and DPPH radicals, as well as SOD activity and FRAP. Polyphenols and flavonoids have been identified as the main antioxidant ingredients of *Sanghuangporus* [30,81,82]. However, the antioxidant activity of polyphenols in this study is not as high as that of flavonoids. One reason for this discrepancy may be that the antioxidant activity of polyphenols is related to the number and position of phenolic hydroxyl groups [84]. Triterpenoids are regarded as one of the most important bioactive ingredients in *Ganoderma* with multiple biological activities [57,58,59]. In the present investigation, the triterpenoid content is correlated positively with SOD activity but negatively with superoxide radical scavenging activity, revealing that triterpenoid is not the main active component in *Sanghuangporus* responsible for its antioxidant function. AA has a very strong antioxidant capacity and is often utilized as a positive reference in antioxidant tests. This study indicates that AA is one of the main antioxidant active components in *Sanghuangporus*, which is significantly correlated with FRAP and scavenging activities against superoxide and DPPH radicals. According to the results from the correlation analysis, the antioxidant capacities of *Sanghuangporus* are mainly related to the contents of flavonoid and AA, followed by polyphenol and triterpenoid, and finally, polysaccharide. Currently, some studies have indicated that there are a large number of genes involved in the synthesis/pathway of polysaccharide, polyphenol, flavonoid, triterpenoid, and AA detected in *Sanghuangporus* species [85,86,87,88,89,90,91]. Subsequent studies are still needed to explore genes involved in the correlations between bioactive ingredients and antioxidant activities.

## 4. Conclusions

The main purpose of the current study was to comprehensively and systematically compare the active ingredients and biological activities of 15 wild strains from 8 species of *Sanghuangporus*. Together, the results regarding mycelial biomass; polysaccharide, polyphenol, flavonoid, triterpenoid, and AA contents; scavenging activities against hydroxyl, superoxide, DPPH, and ABTS radicals; SOD activity; and FRAP indicate differences in various indicators among all the strains examined. The strains with stronger activities were identified as *S. baumii* Cui 3573, *S. sanghuang* Cui 14419 and Cui 14441, *S. vaninii* Dai 9061, and *S. zonatus* Dai 10841. The correlation analysis further revealed that the antioxidant activities of these *Sanghuangporus* species are mainly related to the flavonoid and AA contents, followed by polyphenol and triterpenoid, and finally, polysaccharide. The findings of this research contribute further potential resources and information on the bioactivities of *Sanghuangporus,* as well as insights and guidance into the selection of strains for different purposes, with a view toward facilitating the development of bioactive therapeutic agents from wild *Sanghuangporus* species and identifying optimal candidates for artificial cultivation.

## Figures and Tables

**Table 1:** Detailed information on strains, collection sites, and hosts of the *Sanghuangporus* species used in the present study.

Species	Strain	Collection Site	Host
*Sanghuangporus alpinus*	Cui 12444	Mugecuo Scenic Spot, Kangding, Sichuan Province, China	Living tree of *Lonicera* sp.
	Cui 12456	Yading Nature Reserve, Daocheng County, Sichuan Province, China	Dead tree of *Lonicera* sp.
	Cui 17052	Yunshanping, Yulong Snow Mountain, Lijiang, Yunnan Province, China	Living tree of *Lonicera* sp.
*Sanghuangporus baumii*	Cui 3573	Changbai Mountain, Antu County, Jilin Province, China	Fallen angiosperm trunk
	Dai 13331	Botanical Garden, Beijing, China	Living tree of *Syringa* sp.
*Sanghuangporus lonicericola*	Dai 8375	Jingpo Lake, Mudanjiang, Heilongjiang Province, China	Living tree of *Lonicera* sp.
	Dai 17304	Beiling Park, Shenyang, Liaoning Province, China	Living tree of *Lonicera* sp.
*Sanghuangporus quercicola*	Wei 7575	Baotianman Nature Reserve, Neixiang County, Nanyang, Henan Province, China	Fallen wood of *Quercus* sp.
*Sanghuangporus sanghuang*	Cui 14419	Wuai Village, Chihe Town, Shiquan County, Ankang, Shanxi Province, China	Living tree of *Morus* sp.
	Cui 14441	Pipahe Village, Duohuo Town, Lingchuan County, Jincheng, Shanxi Province, China	Living tree of *Morus* sp.
*Sanghuangporus vaninii*	Dai 8236	Changbai Mountain, Antu County, Jilin Province, China	Living tree of *Populus* sp.
	Dai 8245	Changbai Mountain, Antu County, Jilin Province, China	Living tree of *Populus* sp.
	Dai 9061	Changbai Mountain, Antu County, Jilin Province, China	Fallen angiosperm trunk
*Sanghuangporus weigelae*	Dai 15768	Jinfoshan Forest Park, Nanchuan County, Chongqing, China	Dead tree of *Weigela* sp.
*Sanghuangporus zonatus*	Dai 10841	Jianfengling Nature Reserve, Ledong County, Hainan Province, China	Fallen angiosperm trunk

## Data Availability

The original contributions presented in this study are included in the article/Supplementary material. Further inquiries can be directed to the corresponding author.

## Supplementary Materials

The following supporting information can be downloaded at https://www.mdpi.com/article/10.3390/jof9020242/s1, Table S1: Variations in bioactive ingredients and antioxidant activities during submerged fermentation of 15 wild strains from 8 species of *Sanghuangporus*.
