# Supplementary material for: Identifying Bioactive Ingredients and Antioxidant Activities of Wild Sanghuangporus Species of Medicinal Fungi
Source: J Fungi (Basel). 2023 Feb 11;9(2):242. doi: 10.3390/jof9020242 (PMC9959451; doi:10.3390/jof9020242)
Supplement: Supplementary file 1 [file jof-09-00242-s001.zip › jof-2201729-supplementary.pdf]

Table S1. Variations in bioactive ingredients and antioxidant activities during submerged fermentation of 15 wild strains from 8 species of *Sanghuangporus*.

Indicator	Species	Strain number	Incubation period						
			Day 2	Day 4	Day 6	Day 8	Day 10	Day 12	Day 14
Mycelial biomass (g/L)	*Sanghuangporus alpinus*	Cui 12444	0.493±0.009 f	2.112±0.039 e	3.144±0.001 d	3.786±0.231 c	4.664±0.001 b	4.744±0.082 b	5.262±0.001 a
		Cui 12456	0.099±0.003 e	0.283±0.033 e	0.696±0.555 d	1.279±0.057 c	1.562±0.119 bc	1.893±0.007 ab	2.203±0.095 a
		Cui 17052	0.064±0.010 f	0.458±0.038 e	1.394±0.109 d	2.039±0.077 c	2.392±0.107 b	2.498±0.021 b	2.718±0.062 a
	Sanghuangporus baumii	Dai 13331	0.805±0.023 d	2.132±0.055 c	3.496±0.198 b	4.036±0.030 a	4.258±0.116 a	4.226±0.157 a	4.203±0.060 a
		Cui 3573	0.429±0.033 f	0.954±0.051 e	2.111±0.082 d	2.871±0.262 c	3.493±0.481 b	3.698±0.197 b	4.647±0.413 a
	Sanghuangporus lonicericola	Dai 17304	0.454±0.000 f	1.618±0.127 e	3.926±0.001 d	4.044±0.082 c	4.096±0.056 c	4.619±0.001 b	5.093±0.001 a
		Dai 8375	0.717±0.001 f	2.549±0.012 e	3.523±0.012 d	4.077±0.021 c	4.322±0.070 b	4.757±0.246 a	4.864±0.181 a
	Sanghuangporus quercicola	Wei 7575	1.022±0.087 e	2.522±0.141 d	3.904±0.186 c	4.354±0.789 bc	4.683±0.010 ab	5.213±0.093 ab	4.954±0.031 a
	Sanghuangporus sanghuang	Cui14419	0.176±0.012 g	0.608±0.019 f	2.136±0.304 e	3.130±0.080 d	4.296±0.057 c	4.836±0.267 b	5.404±0.405 a
		Cui 14441	0.683±0.088 e	1.202±0.200 d	2.160±0.160 c	2.524±0.076 c	3.042±0.002 b	3.243±0.621 b	3.833±0.154 a
	Sanghuangporus vaninii	Dai 8236	0.355±0.201 e	0.718±0.031 d	1.050±0.254 c	1.318±0.090 c	1.651±0.214 b	1.912±0.094 b	2.346±0.190 a
		Dai 8245	0.373±0.022 g	0.742±0.043 f	1.474±0.003 e	2.050±0.000 d	2.280±0.070 c	2.968±0.053 b	3.259±0.092 a
		Dai 9061	0.358±0.043 e	1.801±0.080 d	4.125±0.278 c	5.519±0.668 b	6.116±0.499 ab	5.804±0.285 a	4.951±0.093 a
	Sanghuangporus weigelae	Dai 15768	0.431±0.022 f	0.575±0.087 f	0.930±0.031 e	1.306±0.057 d	1.596±0.104 c	2.559±0.121 b	3.388±0.132 a
	Sanghuangporus zonatus	Dai 10841	0.697±0.099 g	1.336±0.027 f	3.259±0.143 e	3.481±0.049 d	4.173±0.137 c	5.104±0.134 b	5.706±0.095 a
Polysaccharide content (mg/mL)	*Sanghuangporus alpinus*	Cui 12444	0.067±0.040 c	0.200±0.026 b	0.300±0.061 ab	0.300±0.069 ab	0.333±0.100 ab	0.433±0.058 a	0.333±0.091 ab
		Cui 12456	0.433±0.057 c	0.467±0.169 bc	0.500±0.115 bc	0.600±0.113 bc	0.767±0.101 ab	0.950±0.231 a	0.667±0.120 bc
		Cui 17052	0.333±0.068 a	0.500±0.044 a	0.667±0.211 a	0.700±0.151 a	0.867±0.137 a	0.900±0.137 a	0.933±0.153 a
	Sanghuangporus baumii	Dai 13331	0.267±0.100 b	0.400±0.110 b	0.567±0.134 ab	0.667±0.134 ab	0.567±0.191 ab	0.900±0.200 a	0.850±0.075 a
		Cui 3573	0.667±0.127 a	0.733±0.060 a	0.933±0.389 a	0.933±0.169 a	1.033±0.153 a	1.000±0.147 a	1.000±0.221 a
	Sanghuangporus lonicericola	Dai 17304	0.233±0.051 b	0.667±0.058 a	0.433±0.072 ab	0.433±0.100 ab	0.367±0.055 b	0.500±0.148 ab	0.650±0.127 a

		Dai 8375	0.333±0.092 ab	0.233±0.068 b	0.400±0.131 ab	0.433±0.060 ab	0.633±0.118 a	0.550±0.188 ab	0.667±0.058 a
	Sanghuangporus quercicola	Wei 7575	0.433±0.105 c	0.567±0.131 bc	0.600±0.070 bc	0.633±0.095 bc	0.667±0.110 bc	0.900±0.085 ab	1.000±0.200 a
	Sanghuangporus sanghuang	Cui 14419	0.667±0.185 b	0.800±0.0845 ab	0.867±0.095 ab	1.033±0.159 ab	0.933±0.258 ab	0.900±0.168 ab	1.233±0.252 a
		Cui 14441	0.800±0.171 bc	0.567±0.091 c	0.533±0.038 c	0.833±0.075 bc	1.167±0.179 ab	1.133±0.332 ab	1.366±0.153 a
	Sanghuangporus vaninii	Dai 8236	0.467±0.075 a	0.600±0.044 a	0.766±0.588 a	0.800±0.148 a	0.800±0.166 a	0.833±0.578 a	0.800±0.155 a
		Dai 8245	0.333±0.092 b	0.467±0.085 b	0.50±0.066 b	0.500±0.142 b	0.567±0.200 b	0.667±0.155 b	1.133±0.115 a
		Dai 9061	0.200±0.026 b	0.467±0.136 ab	0.400±0.095 ab	0.467±0.140 ab	0.500±0.148 ab	0.633±0.115 a	0.633±0.210 a
	Sanghuangporus weigelae	Dai 15768	0.167±0.060 c	0.667±0.230 b	0.500±0.151 b	0.733±0.118 b	0.800±0.135 b	1.067±0.175 a	1.300±0.100 a
	Sanghuangporus zonatus	Dai 10841	0.300±0.125 c	0.233±0.153 c	0.667±0.115 a	0.667±0.196 a	0.367±0.093 bc	0.233±0.153 c	0.567±0.050 ab
Polyphenol content (μg/mL)	*Sanghuangporus alpinus*	Cui 12444	1.821±0.002 c	2.39±0.01 b	2.605±0.012 ab	2.999±0.018 a	3.066±0.03 a	2.925±0.008 a	2.383±0.004 b
		Cui 12456	2.879±0.007 ab	3.334±0.078 a	1.586±0.007 b	1.807±0.013 bc	1.76±0.031 bc	2.269±0.008 abc	2.738±0.021 abc
	Sanghuangporus baumii	Cui 17052	3.455±0.211 e	4.533±0.002 c	3.97±0.004 d	5.035±0.016 b	5.906±0.01 a	2.966±0.016 f	6.040±0.031 a
		Dai 13331	3.87±0.014 b	7.276±0.001 a	7.225±0.032 a	8.079±0.033 a	7.235±0.009 a	7.577±0.003 a	7.045±0.03 a
		Cui 3573	3.428±0.054 b	6.763±0.112 ab	9.405±0.045 a	6.964±0.067 ab	6.569±0.093 ab	8.84±0.133 a	9.154±0.173 a
	Sanghuangporus lonicericola	Dai 17304	6.171±0.056 ab	6.482±0.044 ab	6.432±0.001 ab	6.803±0.259 ab	4.654±0.01 b	5.913±0.021 ab	9.663±0.114 a
		Dai 8375	7.058±0.04 ab	7.534±0.038 ab	5.772±0.034 b	9.523±0.114 a	9.054±0.022 ab	8.17±0.139 ab	5.779±0.132 b
	Sanghuangporus quercicola	Wei 7575	5.608±0.005 a	4.915±0.117 a	4.834±0.027 a	5.792±0.022 a	5.859±0.013 a	5.457±0.024 a	5.504±0.013 a
	Sanghuangporus sanghuang	Cui 14419	5.025±0.088 a	4.322±0.1ab	5.156±0.022 a	5.390±0.023 a	4.995±0.026 a	3.177±0.003 b	4.423±0.066 ab
		Cui 14441	7.909±0.002 a	7.698±0.037 a	4.828±0.027 cd	7.118±0.055 ab	5.645±0.032 bc	4.988±0.112 cd	3.569±0.018 d
	Sanghuangporus vaninii	Dai 8236	6.629±0.068 b	9.248±0.14 b	12.758±0.133 a	10.105±0.044 b	9.482±0.097 b	8.451±0.026 b	8.883±0.017 b
		Dai 8245	6.857±0.076 b	7.641±0.11 b	7.882±0.114 b	11.445±0.057	12.017±0.056	11.706±0.016 a	11.833±0.003 a

						a	a		
		Dai 9061	2.658±0.067 d	4.613±0.133 cd	6.874±0.027 bc	7.165±0.192 bc	7.734±0.113 bc	11.194±0.019 ab	12.238±0.054 a
	Sanghuangporus weigelae	Dai 15768	4.667±0.13 a	4.721±0.101 a	5.397±0.153 a	6.964±0.123 a	4.721±0.049 a	4.386±0.113 a	6.954±0.06 a
	Sanghuangporus zonatus	Dai 10841	2.457±0.071 b	3.234±0.07 b	4.721±0.157 ab	6.837±0.214 ab	7.801±0.118 ab	8.083±0.286 ab	10.567±0.175 a
Flavonoid content (mg/mL)	*Sanghuangporus alpinus*	Cui 12444	2.390±0.004 b	2.410±0.005 b	2.510±0.006 b	2.470±0.006 b	2.771±0.009 a	2.390±0.003 b	2.430±0.004 b
		Cui 12456	2.430±0.004 b	2.410±0.004 b	2.410±0.003 b	2.751±0.010 a	2.610±0.008 ab	2.530±0.006 ab	2.410±0.003 b
		Cui 17052	5.120±0.000 c	5.181±0.003 c	5.161±0.001 c	6.325±0.008 bc	8.293±0.037 ab	7.189±0.005 abc	8.996±0.014 a
	Sanghuangporus baumii	Dai 13331	5.422±0.001 b	6.928±0.012 ab	6.378±0.051 ab	8.414±0.006 ab	8.876±0.002 a	9.197±0.015 a	8.574±0.014 ab
		Cui 3573	4.578±0.009 d	8.655±0.010 d	14.277±0.029 c	18.996±0.036 bc	23.273±0.018 b	25.000±0.043 b	35.261±0.118 a
	Sanghuangporus lonicericola	Dai 17304	5.161±0.004 a	6.365±0.004 a	9.498±0.048 a	9.277±0.020 a	9.398±0.051 a	8.514±0.003 a	8.775±0.047 a
		Dai 8375	3.795±0.004 b	3.675±0.001 b	3.735±0.002 b	7.450±0.066 ab	7.801±0.012 a	9.458±0.011 a	10.723±0.022 a
	Sanghuangporus quercicola	Wei 7575	4.819±0.005 d	10.783±0.017 c	20.120±0.068 b	22.008±0.031 b	22.028±0.037 b	31.124±0.113 ab	25.763±0.044 a
	Sanghuangporus sanghuang	Cui 14419	3.133±0.001 c	4.739±0.001 c	10.402±0.004 b	11.627±0.014 b	16.145±0.026 ab	18.795±0.140 a	21.044±0.333 a
		Cui 14441	7.229±0.003 e	9.257±0.004 de	11.345±0.010 d	15.361±0.026 c	17.731±0.034 bc	18.815±0.016 b	22.430±0.044 a
	Sanghuangporus vaninii	Dai 8236	5.100±0.002 b	5.261±0.003 b	5.884±0.006 ab	6.024±0.012 ab	5.863±0.005 ab	6.546±0.013 a	6.606±0.007 a
		Dai 8245	4.980±0.002 b	4.960±0.004 b	5.000±0.002 b	5.502±0.007 b	6.124±0.013 b	8.474±0.046 a	8.855±0.009 a
		Dai 9061	5.261±0.002 a	5.080±0.001 a	5.161±0.002 a	5.100±0.003 a	4.940±0.001 a	5.221±0.007 a	5.000±0.001 a
	Sanghuangporus weigelae	Dai 15768	5.261±0.001 b	6.205±0.008 b	6.908±0.032 b	7.108±0.018 b	7.610±0.018 b	10.281±0.018 b	15.823±0.100 a
	Sanghuangporus zonatus	Dai 10841	4.940±0.001 d	5.060±0.003 d	7.289±0.026 cd	10.181±0.040 bc	11.265±0.048 ab	13.976±0.023 ab	14.699±0.038 a
Triterpenoid content (μg/mL)	*Sanghuangporus alpinus*	Cui 12444	11.332±0.093 a	6.352±0.066 b	4.507±0.007 bc	3.039±0.021 c	2.047±0.039 c	1.531±0.009 c	1.521±0.010 c
		Cui 12456	4.547±0.056 a	3.485±0.016	1.977±0.020 b	1.779±0.019 b	1.689±0.022 b	1.352±0.012 b	1.898±0.031 b

				ab					
		Cui 17052	2.483±0.006 ab	4.031±0.083 a	1.848±0.023 b	0.559±0.003 b	0.747±0.005 b	0.678±0.006 b	0.846±0.008 b
	Sanghuangporus baumii	Dai 13331	4.487±0.049 a	3.564±0.005 ab	3.287±0.023 ab	2.275±0.006 b	2.443±0.010 b	3.197±0.015 ab	3.693±0.012 ab
		Cui 3573	4.110±0.043 a	3.693±0.015 a	3.068±0.018 a	3.892±0.032 a	3.406±0.018 a	3.723±0.023 a	6.045±0.073 a
	Sanghuangporus lonicericola	Dai 17304	4.070±0.048 a	3.019±0.028 ab	2.047±0.026 bc	0.658±0.005 c	0.697±0.005 c	0.618±0.001 c	0.658±0.001 c
		Dai 8375	12.255±0.083 a	8.465±0.105 ab	7.314±0.091 abc	6.759±0.113 abc	4.031±0.070 bc	1.174±0.013 c	5.311±0.097 bc
	Sanghuangporus quercicola	Wei 7575	3.525±0.105 a	5.350±0.097 a	6.511±0.127 a	6.699±0.012 a	4.160±0.113 a	6.977±0.050 a	8.535±0.037 a
	Sanghuangporus sanghuang	Cui 14419	11.908±0.071 a	11.744±0.108 a	11.372±0.162 a	11.055±0.088 a	9.100±0.059 a	8.148±0.124 a	4.120±0.049 a
		Cui 14441	10.658±0.055 a	7.622±0.049 b	5.965±0.060 b	5.851±0.021 b	5.360±0.569 b	5.936±0.013 b	3.902±0.056 b
	Sanghuangporus vaninii	Dai 8236	4.388±0.045 c	5.201±0.024 c	7.801±0.032 c	16.809±0.044 b	20.529±0.131 ab	20.449±0.016 ab	22.979±0.075 a
		Dai 8245	4.934±0.023 a	4.735±0.074 a	4.616±0.022 a	4.517±0.071 a	1.878±0.007 a	1.283±0.006 a	3.068±0.020 a
		Dai 9061	8.882±0.013 a	7.910±0.065 a	0.896±0.010 b	0.816±0.013 b	0.658±0.011 b	0.360±0.011 b	0.539±0.007 b
	Sanghuangporus weigelae	Dai 15768	6.580±0.031 a	8.158±0.055 a	11.898±0.135 a	11.982±0.069 a	14.438±0.177 a	10.053±0.147 a	8.078±0.028 a
	Sanghuangporus zonatus	Dai 10841	19.914±0.108 a	18.287±0.154 a	12.354±0.092 b	8.842±0.140 bc	3.545±0.032 c	3.168±0.025 c	2.815±0.012 c
Ascorbic acid content (μg/mL)	*Sanghuangporus alpinus*	Cui 12444	5.803±0.006 a	11.831±0.011 a	10.141±0.019 a	11.268±0.005 a	11.887±0.015 a	11.831±0.040 a	7.437±0.008 a
		Cui 12456	4.789±0.006 c	3.042±0.010 c	1.803±0.004 c	3.662±0.006 c	7.859±0.011 bc	13.521±0.012 ab	20.113±0.051 a
		Cui 17052	8.394±0.048 cd	9.014±0.008 cd	6.704±0.006 d	14.704±0.009 bcd	16.845±0.016 abc	20.451±0.009 ab	24.282±0.031 a
	Sanghuangporus baumii	Dai 13331	3.944±0.004 c	16.394±0.028 b	21.352±0.012 ab	25.972±0.022 a	25.070±0.003 a	22.535±0.026 ab	19.211±0.024 ab
		Cui 3573	11.155±0.018 c	14.761±0.010 c	20.507±0.018 bc	32.535±0.015 a	28.958±0.026 ab	30.986±0.026 ab	33.577±0.008 a
	Sanghuangporus lonicericola	Dai 17304	4.789±0.008 b	14.366±0.022 a	19.437±0.029 a	17.577±0.003 a	19.549±0.004 a	17.746±0.004 a	14.620±0.001 a

		Dai 8375	5.859±0.004 b	4.563±0.004 b	5.352±0.008 b	31.606±0.077 a	28.479±0.006 a	25.690±0.000 a	19.493±0.012 ab
	Sanghuangporus quercicola	Wei 7575	18.254±0.006 ab	21.972±0.011 a	18.366±0.009 ab	15.099±0.002 b	17.239±0.003 b	17.465±0.003 b	19.014±0.016 ab
	Sanghuangporus sanghuang	Cui 14419	6.648±0.006 c	8.732±0.025 c	24.056±0.033 bc	39.775±0.139 b	70.648±0.075 a	82.817±0.072 a	43.775±0.016 b
		Cui 14441	10.141±0.003 c	12.958±0.005 c	33.408±0.024 b	42.423±0.024 a	50.366±0.007 a	50.704±0.017 a	50.366±0.037 a
	Sanghuangporus vaninii	Dai 8236	6.986±0.001 d	8.676±0.003 d	11.437±0.012 c	16.845±0.009 ab	15.042±0.004 b	18.648±0.011 a	16.113±0.009 ab
		Dai 8245	6.028±0.003 c	8.169±0.003 c	6.761±0.008 c	9.690±0.012 bc	10.310±0.006 bc	17.915±0.040 ab	20.789±0.046 a
		Dai 9061	18.704±0.020 a	13.268±0.023 b	5.408±0.001 c	6.761±0.008 c	4.056±0.012 c	2.535±0.005 c	5.070±0.025 c
	Sanghuangporus weigelae	Dai 15768	6.197±0.020 c	3.944±0.004 c	3.887±0.003 c	19.380±0.063 bc	23.662±0.041 bc	45.803±0.075 b	72.845±0.177 a
	Sanghuangporus zonatus	Dai 10841	5.521±0.020 b	7.042±0.015 b	36.620±0.064 ab	43.718±0.081 ab	49.972±0.036 a	67.324±0.151 a	77.690±0.172 a
Hydroxyl radicals scavenging activity (U/mL)	*Sanghuangporus alpinus*	Cui 12444	63.848±0.000 b	78.160±0.051 a	71.194±0.001 ab	71.979±0.010 ab	73.833±0.004 ab	70.409±0.008 ab	74.760±0.043 ab
		Cui 12456	88.240±0.005 a	88.097±0.000 a	85.340±0.004 a	71.313±0.041 ab	62.421±0.107 ab	47.301±0.080 b	45.304±0.204 b
		Cui 17052	84.531±0.008 a	83.485±0.019 a	78.825±0.013 a	82.059±0.020 a	79.967±0.033 a	81.726±0.041 a	81.108±0.026 a
	Sanghuangporus baumii	Dai 13331	102.267±0.005 a	102.124±0.008 a	98.986±0.016 a	99.747±0.003 a	98.510±0.025 a	97.227±0.037 a	89.334±0.007 b
		Cui 3573	82.724±0.014 a	80.727±0.008 a	71.360±0.082 ab	75.687±0.024 ab	75.973±0.006 ab	73.548±0.028 ab	65.417±0.044 b
	Sanghuangporus lonicericola	Dai 17304	84.293±0.013 b	93.375±0.013 a	92.805±0.006 a	92.614±0.017 a	95.753±0.011 a	85.815±0.004 b	91.331±0.011 a
		Dai 8375	101.316±0.010 a	101.221±0.008 a	92.472±0.023 b	94.849±0.032 ab	96.656±0.013 ab	92.448±0.006 b	91.616±0.021 b
	Sanghuangporus quercicola	Wei 7575	55.812±0.017 a	54.481±0.007 ab	47.872±0.043 abc	50.439±0.028 abc	46.350±0.019 bc	44.353±0.017 c	47.206±0.025 abc
	Sanghuangporus sanghuang	Cui 14419	56.620±0.059 a	60.900±0.063 a	64.941±0.076 a	65.607±0.077 a	66.320±0.067 a	60.662±0.018 a	56.288±0.007 a

	Sanghuangporus vaninii	Cui 14441	87.860±0.028 b	89.857±0.025 b	96.799±0.008 a	97.417±0.006 a	97.084±0.002 a	93.470±0.018 ab	93.423±0.012 ab
		Dai 8236	83.438±0.008 a	82.724±0.010 a	81.773±0.010 a	80.204±0.001 a	82.059±0.004 a	82.962±0.001 a	79.634±0.021 a
		Dai 8245	88.620±0.008 a	87.955±0.006 a	88.668±0.005 a	90.142±0.009 a	89.429±0.006 a	86.291±0.025 a	90.285±0.017 a
		Dai 9061	100.698±0.003 b	104.026±0.001 a	105.752±0.022 a	104.406±0.002 a	104.169±0.004 a	103.408±0.010 a	104.121±0.004 a
	Sanghuangporus weigelae	Dai 15768	87.527±0.005 a	87.955±0.005 a	87.028±0.002 a	83.390±0.011 bc	82.724±0.002 c	85.815±0.005 ab	85.482±0.016 ab
	Sanghuangporus zonatus	Dai 10841	95.467±0.022 a	100.317±0.009 a	95.182±0.023 a	90.665±0.036 ab	79.491±0.081 b	81.013±0.029 b	79.063±0.048 b
Superoxide radicals scavenging activity (U/L)	*Sanghuangporus alpinus*	Cui 12444	55.581±0.054 a	66.744±0.007 a	71.337±0.025 a	79.884±0.030 a	49.186±0.017 a	77.791±0.004 a	69.186±0.046 a
		Cui 12456	67.093±0.036 b	52.326±0.008 b	74.302±0.072 b	76.977±0.055 b	112.674±0.057 a	69.767±0.015 b	90.814±0.024 ab
		Cui 17052	75.000±0.018 a	69.593±0.006 a	68.372±0.017 a	67.209±0.015 a	65.930±0.025 a	81.395±0.022 a	85.930±0.015 a
	Sanghuangporus baumii	Dai 13331	94.360±0.026 a	117.791±0.001 a	119.767±0.004 a	122.093±0.008 a	100.814±0.056 a	119.302±0.007 a	76.860±0.094 a
		Cui 3573	68.837±0.024 a	67.674±0.013 a	62.093±0.012 a	73.721±0.038 a	76.860±0.050 a	73.023±0.056 a	71.628±0.048 a
	Sanghuangporus lonicericola	Dai 17304	76.512±0.041 c	109.651±0.024 b	132.093±0.016 a	129.186±0.018 a	126.860±0.027 a	130.698±0.007 a	128.256±0.014 a
		Dai 8375	45.581±0.005 d	80.349±0.013 abc	84.186±0.024 ab	87.093±0.014 a	66.860±0.007 c	68.895±0.004 bc	79.070±0.032 abc
	Sanghuangporus quercicola	Wei 7575	57.558±0.011 a	53.256±0.131 a	58.605±0.006 a	89.477±0.026 a	98.721±0.024 a	93.953±0.024 a	86.686±0.001 a
	Sanghuangporus sanghuang	Cui 14419	77.616±0.009 a	69.535±0.027 a	75.000±0.019 a	78.488±0.009 a	75.930±0.021 a	78.023±0.021 a	76.744±0.023 a
		Cui 14441	67.558±0.147 bc	57.093±0.085 bc	41.977±0.027 c	128.837±0.009 a	136.977±0.006 a	106.512±0.101 ab	135.465±0.035 a
	Sanghuangporus vaninii	Dai 8236	85.698±0.027 a	70.814±0.010 ab	66.977±0.012 ab	64.012±0.006 ab	54.070±0.047 b	47.558±0.033 b	55.581±0.038 b
		Dai 8245	85.233±0.083 a	68.605±0.120 a	69.884±0.108 a	48.488±0.115 a	81.628±0.096 a	116.047±0.007 a	121.860±0.016 a

		Dai 9061	101.86±0.041 a	80.233±0.021 ab	85.233±0.056 ab	68.837±0.017 ab	51.512±0.044 b	70.349±0.076 ab	78.721±0.067 ab
	Sanghuangporus weigelae	Dai 15768	79.419±0.036 a	73.837±0.066 a	78.488±0.017 a	84.070±0.022 a	78.140±0.022 a	75.174±0.002 a	75.174±0.011 a
	Sanghuangporus zonatus	Dai 10841	57.442±0.069 b	70.698±0.026 ab	83.140±0.043 ab	87.326±0.093 ab	77.907±0.005 ab	81.977±0.039 ab	110.349±0.047 a
DPPH radicals scavenging activity (%)	*Sanghuangporus alpinus*	Cui 12444	45.386±1.796 c	53.296±7.191 bc	68.048±8.220 a	68.832±0.399 a	66.227±4.279 ab	66.541±0.929 ab	66.196±5.726 ab
		Cui 12456	55.242±16.483 a	61.111±6.525 a	21.343±1.071 b	27.119±6.126 b	16.761±2.658 b	34.275±8.789 ab	29.567±0.266 b
		Cui 17052	22.505±5.237 c	30.038±7.590 c	29.881±6.409 c	31.262±4.794 c	54.991±1.130 b	66.729±8.368 a	23.164±2.397 c
	Sanghuangporus baumii	Dai 13331	11.111±0.533 b	76.836±0.799 a	76.146±4.839 a	76.460±0.000 a	60.075±7.990 a	59.196±9.379 a	64.595±0.000 a
		Cui 3573	54.300±10.184 a	77.401±7.579 a	86.064±5.327 a	83.239±2.783 a	92.655±1.346 a	93.296±58.501 a	91.149±98.585 a
	Sanghuangporus lonicericola	Dai 17304	72.065±5.766 ab	73.823±5.709 a	63.214±1.463 c	65.788±1.896 bc	61.707±0.392 c	60.201±3.484 c	57.627±3.809 c
		Dai 8375	68.613±0.784 b	77.024±9.32 ab	85.405±6.791 a	54.896±4.927 c	67.797±7.643 b	78.782±2.744 ab	78.594±3.634 ab
	Sanghuangporus quercicola	Wei 7575	71.846±6.525 a	70.119±7.070 a	31.701±9.666 b	84.652±5.992 a	74.513±9.219 a	78.531±21.040 a	81.450±7.857 a
	Sanghuangporus sanghuang	Cui 14419	78.531±1.358 a	66.039±8.046 a	62.712±8.344 a	59.887±35.607 a	59.510±4.654 a	56.058±5.726 a	40.113±21.357 a
		Cui 14441	75.895±8.583 a	54.708±0.932 a	52.354±9.854 a	51.130±10.520 a	64.721±13.091 a	71.877±5.932 a	68.079±6.326 a
	Sanghuangporus vaninii	Dai 8236	39.36±07.990 bc	46.955±6.525 ab	30.131±2.663 c	45.009±8.656 ab	512.852±6.392 ab	58.757±5.327 a	54.802±0.000 ab
		Dai 8245	67.326±4.394 a	70.056±3.995 a	73.886±1.196 a	67.797±4.708 a	68.801±5.488 a	62.241±40.882 a	44.633±59.125 b
		Dai 9061	87.571±7.871 a	81.858±0.133 ab	67.232±7.186 bc	60.201±1.198 c	72.003±6.320 bc	70.245±5.859 bc	42.059±0.533 d
	Sanghuangporus weigelae	Dai 15768	38.701±13.183 c	62.335±3.593 b	63.842±11.186 b	66.416±5.414 b	57.439±7.374 b	93.315±0.399 a	95.386±1.997 a
	Sanghuangporus zonatus	Dai 10841	33.898±4.528 c	25.612±6.126 c	49.341±5.327 b	61.111±3.063 ab	71.846±9.721 a	78.343±5.126 a	62.429±9.721 ab

ABTS radicals scavenging activity (%)	*Sanghuangporus alpinus*	Cui 12444	78.083±4.002 b	79.538±4.901 b	80.059±2.508 a	92.079±8.631 a	93.729±1.031 a	97.277±2.952 a	95.05±0.990 a
		Cui 12456	99.257±0.350 a	98.515±0.000 a	84.983±7.770 a	92.079±8.631 a	95.505±4.957 a	97.277±2.450 a	96.287±4.551 a
		Cui 17052	58.663±2.450 b	81.518±3.512 a	55.446±18.437 b	93.894±1.031 a	20.297±0.000 cd	40.759±13.579 bc	12.129±10.152 d
	Sanghuangporus baumii	Dai 13331	84.488±4.201 b	95.215±4.001 a	91.914±1.400 ab	95.545±0.857 a	95.380±4.201 a	86.304±1.050 ab	91.914±5.780 ab
		Cui 3573	97.195±1.143 a	95.05±1.310 a	92.739±2.727 a	93.069±4.122 a	94.884±0.756 a	92.739±1.143 a	93.234±0.572 a
	Sanghuangporus lonicericola	Dai 17304	45.462±5.251 b	96.040±3.501 a	99.010±0.700 a	96.700±4.660 a	96.700±7.214 a	97.772±4.002 a	97.277±1.419 a
		Dai 8375	88.284±6.461 a	95.215±1.512 a	94.554±1.400 a	91.749±5.864 a	81.188±9.801 a	75.248±11.059 a	82.178±4.201 a
	Sanghuangporus quercicola	Wei 7575	71.782±1.050 b	88.614±1.152 a	97.195±1.422 a	95.875±3.025 a	87.129±11.343 a	52.805±2.403 c	38.284±2.800 d
	Sanghuangporus sanghuang	Cui 14419	99.010±0.000 a	98.515±0.857 a	98.762±0.350 a	98.845±0.756 a	98.680±0.572 a	98.845±0.756 a	99.670±0.286 a
		Cui 14441	98.185±1.143 a	96.535±0.990 ab	96.370±0.572 ab	94.554±1.400 b	96.700±0.572 ab	98.020±0.000 a	98.845±0.756 a
	Sanghuangporus vaninii	Dai 8236	78.548±3.370 b	98.845±2.061 a	94.884±5.081 a	94.554±2.269 a	97.360±6.461 a	95.710±11.926 a	87.459±4.600 ab
		Dai 8245	95.710±0.756 a	94.389±4.210 a	84.818±5.381 a	81.023±1.782 a	84.158±10.230 a	81.683±5.623 a	88.779±11.613 a
		Dai 9061	85.149±25.724 a	94.059±10.289 a	99.835±0.286 a	96.040±0.000 a	95.875±1.512 a	95.215±0.756 a	98.680±2.287 a
	Sanghuangporus weigelae	Dai 15768	95.050±3.570 a	96.370±3.845 a	96.370±2.340 a	93.564±0.857 a	95.380±1.739 a	91.749±4.959 a	93.399±2.061 a
	Sanghuangporus zonatus	Dai 10841	70.050±0.350 b	93.234±4.032 ab	68.647±11.885 b	76.073±13.873 ab	87.871±8.051 ab	94.059±4.230 ab	97.525±0.561 a
Superoxide dismutase activity (U/mL)	*Sanghuangporus alpinus*	Cui 12444	62.834±0.008 c	72.142±0.012 b	78.899±0.010 a	80.250±0.010 a	62.383±0.010 c	66.737±0.006 c	63.509±0.003 c
		Cui 12456	58.780±0.013 cd	56.303±0.000 d	60.056±0.010 cd	65.911±0.005 bc	70.829±0.009 ab	74.094±0.017 a	60.582±0.028 cd
		Cui 17052	71.279±0.002 d	75.295±0.014 c	71.767±0.004 d	78.974±0.007 b	83.027±0.005 a	83.853±0.007 a	81.226±0.002 ab

	Sanghuangporus baumii	Dai 13331	43.616±0.083 a	42.565±0.072 a	53.900±0.066 a	52.774±0.038 a	59.230±0.032 a	55.101±0.067 a	37.272±0.046 a
		Cui 3573	98.041±0.006 a	97.816±0.017 a	98.342±0.005 a	98.529±0.006 a	98.642±0.003 a	98.529±0.001 a	95.564±0.009 a
	Sanghuangporus lonicericola	Dai 17304	38.286±0.047 a	29.878±0.012 a	34.007±0.028 a	43.240±0.007 a	51.348±0.093 a	53.075±0.037 a	42.264±0.037 a
		Dai 8375	80.010±0.009 b	93.162±0.024 a	90.534±0.004 a	93.312±0.011 a	94.588±0.012 a	96.503±0.008 a	98.567±0.013 a
	Sanghuangporus quercicola	Wei 7575	98.192±0.006 a	96.765±0.026 a	99.843±0.002 a	96.690±0.013 a	98.567±0.005 a	99.543±0.013 a	96.015±0.002 a
	Sanghuangporus sanghuang	Cui 14419	75.445±0.006 a	76.496±0.012 a	79.574±0.013 a	78.298±0.006 a	78.373±0.013 a	77.360±0.012 a	73.531±0.029 a
		Cui 14441	42.339±0.075 a	45.492±0.027 a	43.841±0.064 a	41.514±0.010 a	49.846±0.064 a	55.777±0.023 a	54.951±0.062 a
	Sanghuangporus vaninii	Dai 8236	91.811±0.010 c	94.213±0.002 bc	92.486±0.004 bc	93.762±0.002 bc	95.339±0.005 b	98.417±0.004 a	99.393±0.008 a
		Dai 8245	40.538±0.004 ab	40.425±0.016 ab	56.303±0.049 a	57.654±0.058 a	43.803±0.013 ab	33.556±0.027 b	35.358±0.011 ab
		Dai 9061	76.872±0.023 d	79.574±0.022 cd	89.033±0.021 abc	95.339±0.019 a	91.435±0.010 ab	85.880±0.006 abcd	82.277±0.017 bcd
	Sanghuangporus weigelae	Dai 15768	53.225±0.068 ab	45.192±0.068 b	51.798±0.071 ab	69.815±0.051 ab	72.067±0.016 ab	75.070±0.019 ab	78.598±0.015 a
	Sanghuangporus zonatus	Dai 10841	73.794±0.012 c	78.523±0.014 b	92.862±0.008 a	93.687±0.004 a	93.762±0.007 a	95.489±0.005 a	95.414±0.010 a
Ferric reducing ability of plasma (μmol/L)	*Sanghuangporus alpinus*	Cui 12444	393.234±39.267 a	420.149±17.975 a	311.284±12.447 b	262.489±20.919 b	305.113±19.640 b	318.801±32.478 b	376.532±31.895 a
		Cui 12456	26.248±8.970 d	88.340±1.053 b	13.482±11.957 d	12.915±12.939 d	42.383±22.417 cd	56.603±3.019 c	119.298±12.638 a
		Cui 17052	58.660±2.914 ab	56.532±5.569 ab	12.064±6.319 b	68.766±1.107 a	58.376±3.619 ab	44.936±4.754 ab	23.872±2.859 ab
	Sanghuangporus baumii	Dai 13331	58.234±19.558 ab	37.454±23.607 b	78.376±16.394 ab	91.957±13.390 a	94.830±20.160 a	47.596±9.328 ab	28.305±8.219 b
		Cui 3573	177.241±42.858 c	329.652±14.098 b	359.369±7.949 b	336.000±11.284 b	336.035±22.039 b	451.000±33.917 ab	501.993±46.816 a
	Sanghuangporus lonicericola	Dai 17304	15.965±22.260 b	24.617±15.478 b	423.482±12.474 a	422.064±7.343 a	442.489±17.068 a	408.872±6.464 a	333.553±14.258 a

	Dai 8375	110.574±10.326 c	208.128±5.710 bc	302.418±8.337 ab	393.021±3.761 a	399.830±17.692 a	98.128±7.833 c	52.489±9.562 c
Sanghuangporus quercicola	Wei 7575	81.000±9.027 b	386.745±15.647 a	339.511±24.072 a	412.809±36.258 a	340.787±8.726 a	340.894±4.363 a	366.745±17.452 a
Sanghuangporus sanghuang	Cui 14419	69.723±4.213 d	51.851±0.569 d	151.426±13.841 c	238.021±24.373 b	348.128±0.451 a	351.426±47.542 a	432.369±50.402 a
	Cui 14441	60.078±9.287 c	200.007±19.850 b	238.872±19.270 b	318.234±12.879 a	194.830±48.143 b	36.319±26.780 c	39.404±32.948 c
Sanghuangporus vaninii	Dai 8236	45.468±4.443 a	20.043±7.001 a	17.454±9.969 a	27.596±3.771 a	35.681±7.753 a	143.553±5.357 a	143.340±7.317 a
	Dai 8245	45.787±0.150 bc	37.312±5.695 c	14.298±0.752 d	49.085±2.316 bc	57.383±1.235 b	222.170±2.558 a	52.596±11.284 bc
	Dai 9061	75.149±9.408 cd	181.355±3.536 a	154.050±8.374 ab	130.681±4.771 abc	37.454±3.625 d	101.745±10.080 bcd	129.936±18.640 abc
Sanghuangporus weigelae	Dai 15768	11.745±9.177 c	8.340±7.372 c	23.979±2.356 c	75.965±16.882 b	36.213±14.293 c	90.787±2.407 ab	113.340±0.301 a
Sanghuangporus zonatus	Dai 10841	64.191±50.368 d	65.610±24.534 d	80.149±5.717 d	251.851±43.630 c	456.426±33.249 b	708.553±17.903 a	705.894±14.699 a
